# New seco-anthraquinone glucoside from the roots of *Rumex crispus*

**DOI:** 10.1007/s13659-022-00350-3

**Published:** 2022-08-03

**Authors:** Yong-Xiang Li, Na Li, Jing-Juan Li, Man Zhang, Hong-Tao Zhu, Dong Wang, Ying-Jun Zhang

**Affiliations:** 1 State Key Laboratory of Phytochemistry and Plant Resources in West China, Kunming Institute of Botany, Chinese Academy of Sciences, Kunming, 650204 People’s Republic of China; 2 University of Chinese Academy of Sciences, Beijing, 100049 People’s Republic of China; 3 Yunnan Key Laboratory of Natural Medicinal Chemistry, Kunming Institute of Botany, Chinese Academy of Sciences, Kunming, 650201 People’s Republic of China

**Keywords:** Polygonaceae, *Rumex crispus* L., Anthranoids, Anti-fungal activity

## Abstract

A new seco-anthraquinone, crispuside A (**1**), and three new 3,4-dihydronaphthalen-1(2*H*)-ones, napthalenones A-C (**2**–**4**), were isolated from the roots of *Rumex crispus* L., along with 10 known anthraquinones (**6–14**) and naphthalenone (**5**). Their structures were fully determined by extensive spectroscopic analyses, including ECD, and X-ray crystallography in case of compound **5**, whose absolute configuration was determined for the first time. The isolates **1**, **6**–**14** were evaluated for their anti-inflammatory and anti-fungal activity against three skin fungi, e.g., *Epidermophyton floccosum*, *Trichophyton rubrum*, and *Microsporum gypseum*. Most of the isolates showed weak anti-fungal and anti-inflammatory activity. Only compound **9** exhibited obvious anti-fungal activity against *E. floccosum* (MIC_50_ = 2.467 ± 0.03 μM) and *M. gypseum* (MIC_50_ = 4.673 ± 0.077 μM), while the MIC_50_ values of the positive control terbinafine were 1.287 ± 0.012 and 0.077 ± 0.00258 μM, respectively. The results indicated that simple emodin type anthraquinone is more potential against skin fungi than its oxyglucosyl, *C*-glucosyl and glycosylated seco analogues.

**Graphical Abstract:**

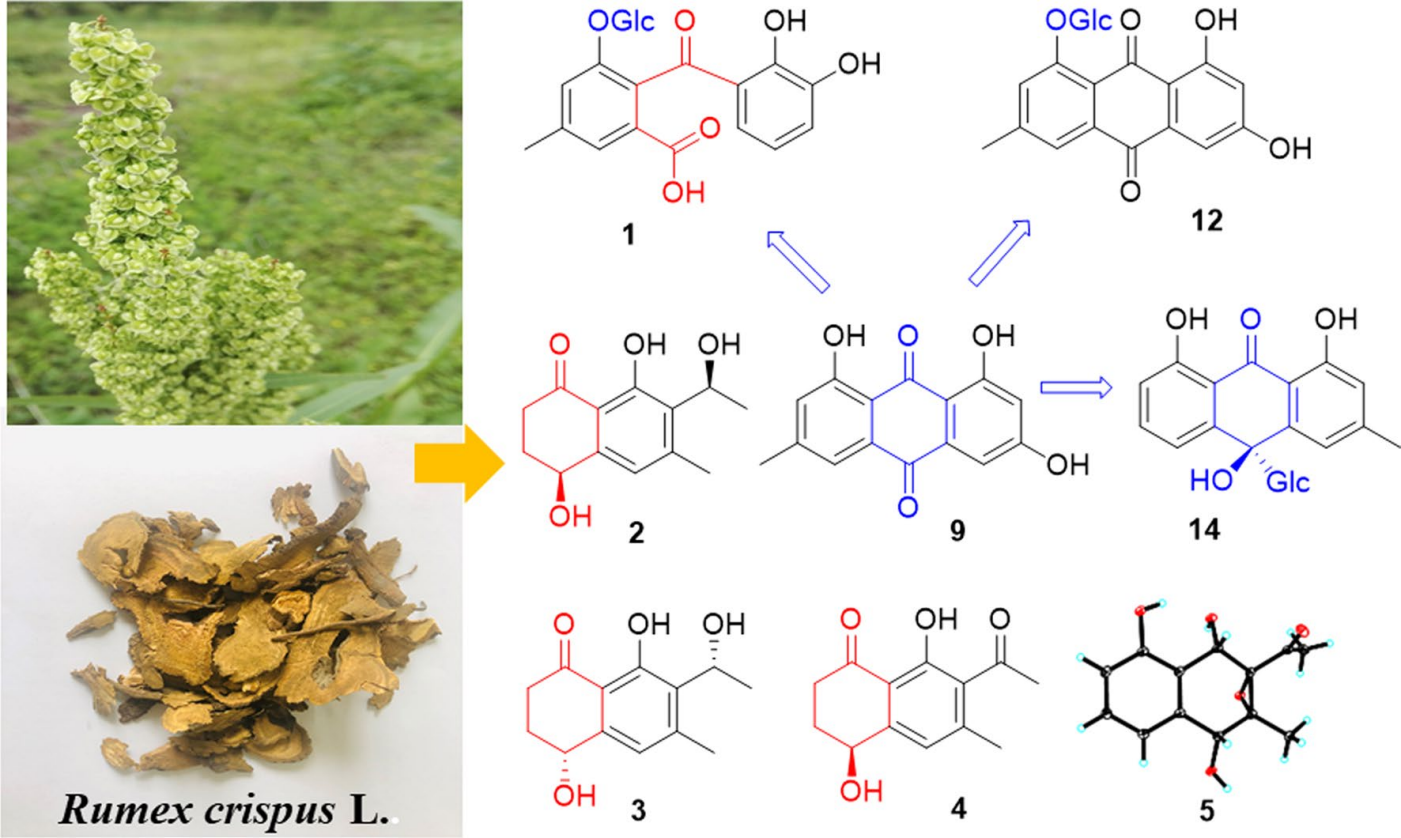

**Supplementary Information:**

The online version contains supplementary material available at 10.1007/s13659-022-00350-3.

## Introduction

The genus *Rumex* with more than 200 species distributing widely in the world is the second largest genus in the family Polygonaceae, in which some species displayed nutritional and medicinal properties. For example, *Rumex patientia* L. was reported having abundant amino acid and cellulose [1]. A series of anthranoids, tannins, naphthalenes, and flavonoids were identified as the major chemical compositions from *Rumex* species [2–5].

**Fig. 1:**
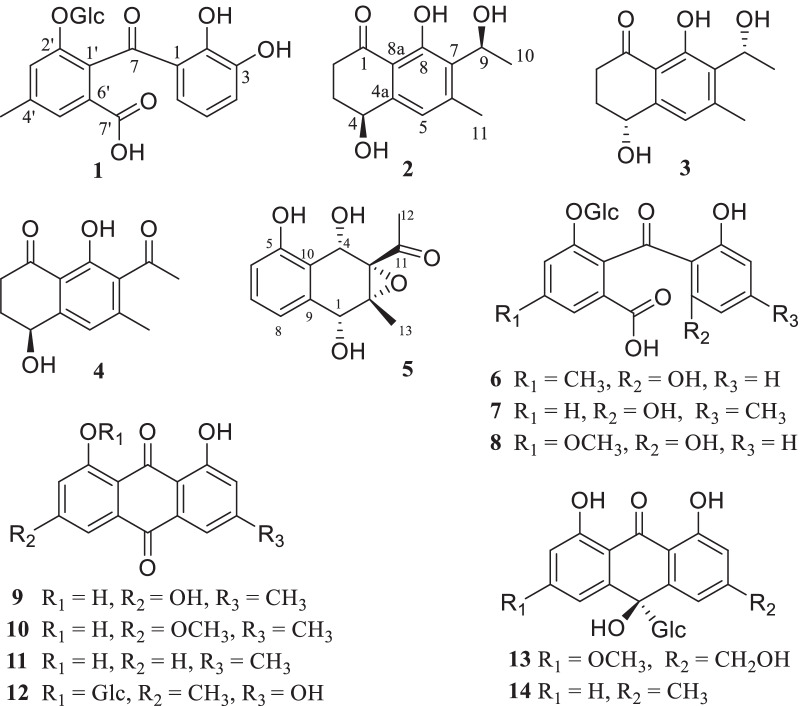
Compounds **1**–**14** isolated from the roots of *Rumex crispus* L.

*Rumex crispus* L., a perennial herbaceous species with stout and straight root system, is widely distributed in China, Korea, Kazakhstan, Russia, Japan, Europe, and North America [6, 7]. It has been used medicinally for treating jaundice and related liver diseases, stomachache, neckache, low blood pressure, pneumonia, wound healing, and rheumatism [8–10]. The crude extract was reported to possess anti-inflammatory, antimicrobial, antioxidant, and anti-diabetic properties [11–14]. However, the chemical compositions are so far not well-known. Our detailed phytochemical study on the roots of *R. crispus* led to the isolation of four new compounds including a seco-anthraquinone glucoside (**1**) and three naphtholones (**2–4**), along with 10 known anthraquinones (**6–14**) and napthalenone (**5**) (Fig. 1). Most of the isolates, **1** and **6**–**14**, were evaluated for their anti-fungal activity against three skin fungi (*Epidermophyton floccosum*, *Trichophyton rubrum*, *Microsporum gypseum*), and the anti-inflammatory properties. Herein, we report the study.

## Result and discussion

The air-dried roots of *R. crispus* were crushed into small grains and extracted with 90% aqueous MeOH. After removal of the organic solvent, the crude extract was suspended into water and fractionated with ethyl acetate. The ethyl acetate fraction was further applied to repeated column chromatography (CC) over Sephadex LH-20, macroporous resin D101, silica gel, and RP-18, followed with semipreparative HPLC, yielded 14 compounds. Among which, one seco-anthraquinone glucoside (**1**) and three naphtholones (**2**–**4**) are new compounds. Ten known compounds were identified as 3-acetyl-2-methyl-1,4,5-trihydroxy-2,3-epoxynaphthoquinol (**5**) [15], nepalensides A (**6**) and B (**7**) [16], polyanthraquinoside A (**8**) [17], emodin (**9**) [18], physcion (**10**) [19], chrysophanol (**11**) [20], emodin-1-*O*-*β*-D-glucopyranoside (**12**) [21], 6-methoxyl-10-hydroxyaloin B (**13**) [22], (10*R*)-3-methyl-1,8,10-trihydroxy-10-D-glucopyranosyl-9(10*H*)-anthracenone (**14**) [23] (Fig. 1), respectively, by comparison of their spectroscopic data with literature values. Seven compounds, **5**–**8** and **12**–**14**, were isolated from *R. crispus* for the first time.

### Structural identification of compounds

**Fig. 2:**
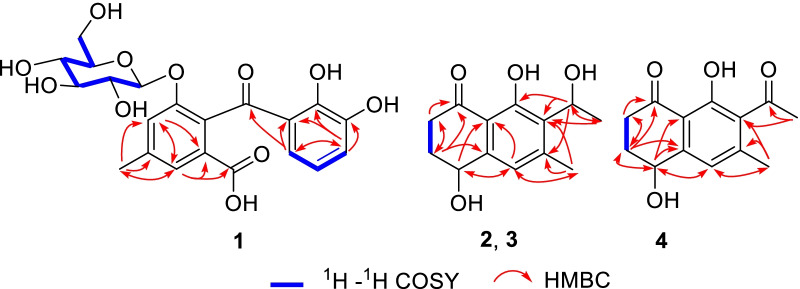
Key ^1^H–^1^H COSY and HMBC correlations of **1**, **2**,** 3** and **4**

Compound **1** was obtained as yellowish amorphous powder. Its molecular formula, C_21_H_22_O_11_, was determined by negative HRESI-TOF–MS (*m/z* 449.1094 [M–H]^−^, calcd. for C_21_H_21_O_11_, 449.1089). In the ^13^C NMR spectrum of **1** (Table 1), 14 carbon signals due to one ketone (*δ*_C_ 203.3), one carboxyl (*δ*_C_ 170.8) and two benzene ring (*δ*_C_ 106.0–165.0, 12 × C) were observed, assignable to a carboxylated benzophenone. In addition, 6 carbon signals at *δ*_C_ 101.3 (C-1″), 74.7 (C-2″), 78.2 (C-3″), 71.7 (C-4″), 77.8 (C-5″), and 62.5 (C-6″) from a glucosyl moiety and a methyl signal at *δ*_C_ 21.4 were also observed. In the ^1^H NMR spectrum of **1**, three characteristic proton resonances at *δ*_H_ 6.61 (1H, d, *J* = 8.3 Hz, H-4), 7.36 (1H, t, *J* = 8.3 Hz, H-5) and 6.56 (1H, d, *J* = 8.3 Hz, H-6) suggested the existence of a 1,2,3-trisubstituted benzene ring [24]. Moreover, two aromatic singlet resonances at *δ*_H_ 6.90 (1H, s, H-3′) and 7.34 (1H, br s, H-5′)] and a 3-H singlet signal at *δ*_H_ 2.38 (3H, s) due to a methyl group were observed, together with one anomeric proton at *δ*_H_ 4.85 (1H, d, *J* = 7.8, H-1″) and a set of proton signals in the range between *δ*_H_ 2.3–3.8, disclosing the existence of a sugar moiety. The *J*_1″−2″_ coupling constant of anomeric proton (7.8 Hz) revealed the glucosyl anomeric center to be *β* configuration. The ^1^H and ^13^C NMR data of **1** were closely related to those of **6** [16]. However, instead of a symmetric 1,2,3-trisubstituted benzene ring in **6**, an un-symmetric 1,2,3-trisubstituted benzene ring appeared in **1**. This was confirmed by the HMBC correlations of H-4 (*δ*_H_ 6.61) with C-1 (*δ*_C_ 114.9), C-2 (*δ*_C_ 165.0), C-3 (*δ*_C_ 160.0) and C-6 (*δ*_C_ 106.0), and H-6 (*δ*_H_ 6.56) with C-1 (*δ*_C_ 114.9), C-4 (*δ*_C_ 112.4) and C-7 (*δ*_C_ 203.3). Moreover, the HMBC correlations from methyl proton at *δ*_H_ 2.38 to C-3′ (*δ*_C_ 121.8), C-4′ (*δ*_C_ 140.8), and C-5′ (*δ*_C_ 122.8), from H-3′ (*δ*_H_ 6.90) to C-5′ and C-6′ (*δ*_C_ 132.3), and from H-5′ (*δ*_H_ 7.34) to C-3′, C-6′ and C-7′ (*δ*_C_ 170.8) (Fig. 2) confirmed the structure of **1**. Therefore, the structure of compound **1** was established as shown in Fig. 1 and named as crispuside A.

**Table 1:** ^1^H (600 MHz) and.^13^C (150 MHz) NMR data of **1** in CD_3_OD (*δ* in ppm, *J* in Hz)

No	*δ*_C_, type	*δ*_H_, mult. (*J*)	No	*δ*_C_, type	*δ*_H_, mult. (*J*)
1	114.9, s		5′	122.8, d	7.34 br s
2	165.0, s		6′	132.3, s	
3	160.0, s		7′	170.8, s	
4	112.4, d	6.61 d (8.3)	Me	21.4, q	2.38 s
5	137.1, d	7.36 t (8.3)	1″	101.3, d	4.85 d (7.8)
6	106.0, d	6.56 d (8.3)	2″	74.7, d	2.45 m
7	203.3, s		3″	78.2, d	3.29 m
1′	130.3, s		4″	71.1, d	3.27 m
2′	154.9, s		5″	77.8, d	3.14 t (9.4)
3′ 4′	121.8, d 140.8, s	6.90 s	6″	62.5, t	a 3.77 dd (12.1, 2.3) b 3.61 dd (12.1, 5.8)

**Fig. 3:**
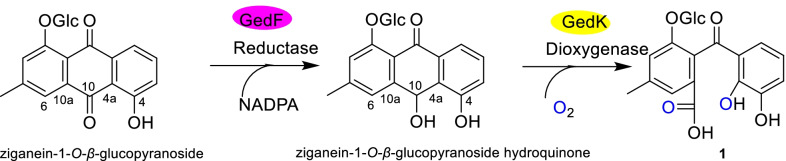
Possible formation of compound **1**

Compound **1** is a new anthraquinone-related compound, whose formation mechanism might be similar to that of desmethylsulochrin, which was established by the ring-opening process of questin catalyzed by GedF (Geodin synthesis protein F) and GedK [25]. In *R. crispus*, compound **1** maybe formed from reduction firstly and then ring-opening of ziganein-1-*O*-*β*-glucopyranoside catalyzed by GedF and GedK respectively (Fig. 3).

**Fig. 4:**
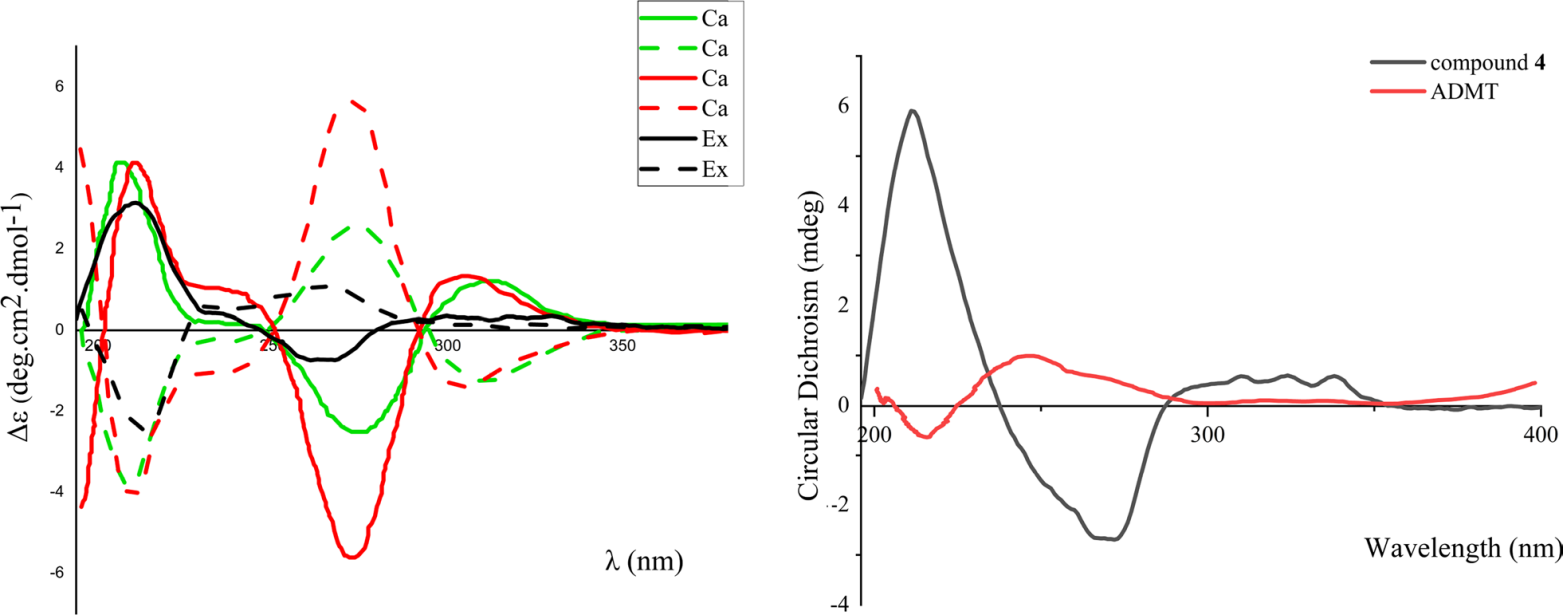
Experimental ECD curves of **2**, **3**, **4** and ADMT, and calculated ECD curves of four stereoisomers of HM

Compounds **2** and **3**, obtained as colorless powder, are a pair of enantiomers possessing the same molecular formula C_13_H_16_O_4_, as deduced by the negative HRESIMS (*m/z* 235.0979 [M−H]^−^ calcd C_13_H_15_O_4_, 235.0976). The ^1^H, ^13^C NMR, and HSQC spectral data of **2** and **3** revealed the existence of two methyls [*δ*_H_ 2.48 (3H, s, H-11), *δ*_C_ 21.1; *δ*_H_ 1.52 (3H, d, *J* = 6.7 Hz, H-10), *δ*_C_ 21.1], two methylenes [*δ*_H_ 2.88 (1H, m, H-2a), 2.67 (1H, m, H-2b), *δ*_C_ 35.9; *δ*_H_ 2.27 (1H, m, H-3a), 2.08 (1H, m, H-3b), *δ*_C_ 32.6], one aromatic methine [*δ*_H_ 6.89 (1H, s, H-5), *δ*_C_ 121.9], two oxymethines [*δ*_H_ (1H, 4.78, dd, *J* = 8.0, 3.8 Hz, H-4), *δ*_C_ 68.1; *δ*_H_ 5.34 (1H, q, *J* = 6.7 Hz, H-9), *δ*_C_ 65.6], one carbonyl group (*δ*_C_ 206.1, C-1), and a set of quaternary aromatic carbons (*δ*_C_ 146.4, 147.2, 130.9, 161.5, 114.6). These spectroscopic features were similar to those of (4*S*,9*S*)-9-hydroxy-*O*-methylasparvenone (4*S*,9*S*-HM) [26], whose molecular weight was 252 Da, 16 Da more than those of **2** and **3**. However, the chemical shifts of C-6 and C-11 in **2** and **3** were obviously different with those of 4*S*,9*S*-HM, indicating that the substituent at C-6 in **2** and **3** was methyl group, instead of a methoxyl group in 4*S*,9*S*-HM. Moreover, the HMBC correlations of H-11 (*δ*_H_ 2.48) with C-5 (*δ*_C_ 121.9), C-6 (*δ*_C_ 147.2) and C-7 (*δ*_C_ 130.9) confirmed the substitution of methyl at C-6 position. The ^1^H–^1^H COSY correlations between H-2 and H-3, and the key HMBC correlations from H-2 to C-1/C-3, from H-3 to C-1/C-2, from H-4 to C-5/C-8a, from H-11 to C-5/C-6/C-7, from H-5 to C-8a, from H-10 to C-7/C-9, from H-9 to C-6/C-7/C-8 determined the planar structures of **2** and **3** as 4,8-dihydroxy-7-(1-hydroxyethyl)-6-methyl-3,4-dihydronaphthalen-1(2*H*)-one. Comparison of the experimental ECD spectrum of **2** with the calculated ECD (Fig. 4) of the four stereoisomers, (4*S*,9*S*)-, (4*R,*9*R*)-, (4*S*,9*R*)- and (4*R*,9*S*)- HM [26] showed the ECD of **2** was more comparable with the computationally derived data for (4*S*,9*S*)- and (4*S*,9*R*)- with positive Cotton effects (CEs) at 248 and 214 nm and negative CE at 280 nm. However, the CE amplitudes of **2** were notably closer to those of the (4*S*,9*S*)- diastereomer, thus favoring the (4*S*,9*S*) absolute configuration for **2**. Therefore, compound **2** was proposed as (4*S*,9*S*)-4,8-dihydroxy-7-(1-hydroxyethyl)-6-methyl-3,4-dihydronaphthalen-1(2*H*)-one. Similarly, the absolute configuration of **3** was deduced as (4*R*,9*R*)-4,8-dihydroxy-7-(1-hydroxyethyl)-6-methyl-3,4-dihydro-naphthalen-1(2*H*)-one. The structures of **2** and **3** were established as shown and named as naphthalenones A (**2**) and B (**3**), respectively.

Compound **4** was isolated as white powder. Its molecular formula was assigned to be C_13_H_14_O_4_, as deduced from the HRESIMS (*m/z* 233.0814 [M−H]^−^, calcd. C_13_H_13_O_4_, 233.0814). The UV spectrum showed absorption bands at *λ*_max_ 286 nm. The 1D and 2D NMR data of **4** (Table 2) indicated it shared the same 3,4-dihydronaphthalen-1(2*H*)-one skeleton with **2**. The molecular weight of **4** is 2 Da less than that of **2**. Comparing the DEPT and ^13^C NMR data of **4** with those of **2** indicated that, a ketone group (*δ*_C_ 206.5) appeared in **4**, instead of an oxymethine C-9 (*δ*_C_ 65.6) in **2**. This was further verified by the HMBC correlation of H-10 with C-9 in the HMBC spectrum of **4**. Comparing with the ECD curve of the known 7-acetyl-4*R*,8-dihydroxy-6-methyl-1-tetralone (ADMT) [27], compound **4** showed a negative CE at 260–270 nm in ECD spectrum, which is in contrast to ADMT (Fig. 4). Therefore, the structure of **4** was determined to be 7-acetyl-4*S*,8-dihydroxy-6-methyl-1-tetralone and named as naphthalenone C.

**Table 2:** ^1^H (600 MHz) and^13^C (150 MHz) NMR data of **2**, **3, 4** and** 5** in CD_3_OD (*δ* in ppm, *J* in Hz)

No	**2**, **3**		**4**^a^		**5**^a^	
	*δ*_C_, type	*δ*_H_ mult. (*J*)	*δ*_C_, type	*δ*_H_ mult. (*J*)	*δ*_C_, type	*δ*_H_ mult. (*J*)
1	206.1, s		205.6, s		70.2, d	4.62 s
2	35.9, t	a 2.88 m b 2.67 m	35.9, t	a 2.82 m b 2.64 m	65.6, s	
3	32.6, t	a 2.27 m b 2.08 m	32.4, t	a 2.25 overlap b 2.03 m	72.6, s	
4	68.1, d	4.78 dd (8.0, 3.8)	67.8, d	4.67 dd (8.7, 3.9)	68.1, d	5.57 s
4a	146.4, s		149.6, s			
5	121.9, d	6.89 s	120.9, d	6.89 s	157.4, s	
6	147.2, s		146.2, s		116.0, d	6.72 d (7.9 Hz)
7	130.9, s		130.4, s		129.9, d	7.17 t (7.9 Hz)
8	161.5, s		160.7, s		119.1, d	7.10 d (7.9 Hz)
8a	114.6, s		114.9, s			
9	65.6, d	5.34 q (6.7)	205.6, d		137.8, s	
10	22.0, q	1.52 d (6.7)	32.2, q	2.49 s	120.2, s	
11	21.1, q	2.48 s	20.5, q	2.25 s	208.6, s	
12					29.1, q	2.36 s
13					15.9, q	1.47 s

^a^Measured at 500 MHz for ^1^H and 125 MHz for ^13^C NMR, respectively

**Fig. 5:**
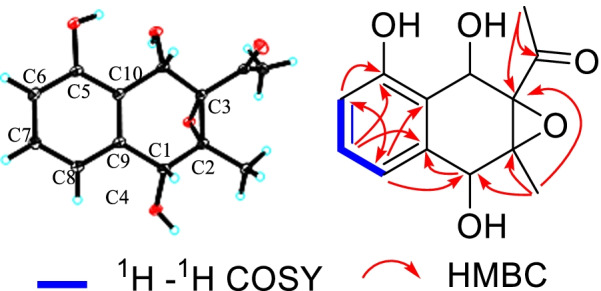
^1^H–^1^H COSY, HMBC spectra and X-ray crystallographic structure of **5**

Compound **5** was isolated as colorless needle crystal with a molecular formula of C_13_H_14_O_5_, as determined by the ESI–MS (negative ion mode) *m/z* 249 [M−H]^−^, and ^13^C NMR and DEPT spectroscopic data. The ^13^C NMR spectrum of **5** showed one carbonyl (*δ*_C_ 208.6), six aromatic carbon signals (*δ*_C_ 157.4, 116.0, 129.9, 119.1, 137.8, 120.2) with three methines and one oxygenated quarternary carbon, and six *sp*^3^ carbon signals (*δ*_C_ 70.2, 65.6, 72.6, 68.1, 29.1, 15.9) assignable to two methyls, two oxymethines and two oxy quarternary carbons. The ^1^H NMR spectrum showed three characteristic proton resonances at *δ*_H_ 6.72 (1H, d, *J* = 7.9 Hz, H-6), 7.17 (1H, t, *J* = 7.9 Hz, H-7), 7.10 (1H, d, *J* = 7.9 Hz, H-8) suggesting the existence of a 5,9,10-trisubstituted benzene ring. In addition, two methyls (*δ*_H_ 2.36, s, H-12; 1.47, s, CH_3_-2) and two oxymethines (6.72, d, *J* = 7.9 Hz, H-6; 5.57, s, H-4) signals were observed. The above data indicated that **5** was a naphthoquinol derivative, whose epoxide ring was inferred by chemical shifts (*δ*_C_ 65.6, C-2; *δ*_C_ 72.6, C-3) and seven degrees of unsaturation. The detailed analyses of the 2D NMR (^1^H-^1^H COSY, HMQC, HMBC) spectra (Fig. 5) deduced the planar structure of **5**, which is the same as the reported 3-acetyl-2-methyl-1,4,5-trihydroxy-2,3-epoxynaphthoquinol without determination of the absolute configuration [15]. In the present study, fine crystal from acetone was obtained and the absolute configuration of **5** was determined by single crystal X-ray diffraction (CDCC Number: 2158643) (Fig. 5). The result confirmed the planar structure of **5**, and revealed unambiguously the absolute configuration as 1*R*, 2*R*, 3*S*, 4*S*.

### Anti-fungal and anti-inflammatory inhibitory activity

Compounds **1** and **6**–**14** were evaluated for their inhibitory effects against three skin fungi (*Epidermophyton floccosum*, *Trichophyton rubrum*, *Microsporum gypseum*) at a concentration of 100 μM (Table S1), as previously described [28], with terbinafine as positive control. Most of them displayed only weak antifungal activity against the three skin fungi, while compound **9** showed obvious antifungal activities against *E. floccosum* and *M. gypseum* with MIC_50_ values of 2.467 ± 0.03 μM and 4.673 ± 0.077 μM, respectively. In case of the antifungal effects against *E. floccosum* and *M. gypseum,* simple emodin type anthraquinone **9** showed the strongest inhibition, followed by oxyglucoside anthraquinone (**12**) or *C*-glucoside oxanthrones (**13**, **14**), and finally glycosylated seco analogues (**1**, **6**–**8**). These compounds (**1**, **6**–**14**) were also evaluated for their anti-inflammatory activity by using LPS to induce the production of iNOS from mouse monocyte macrophage RAW264.7, with L-NMMA as a positive control. The inhibition rate was shown in Table S2, all 10 anthraquinones showed NO inhibitory activity at a concentration of 50 μM, and the order of their inhibition rates is as follows: **9** > **11** > **12** > **13** > **14** > **8** > **10** > **1** > **6** > **7**. Compound **9** had the strongest anti-inflammatory effect, followed by oxyglucoside anthraquinone, *C*-glucoside oxanthrones and finally seco-anthraquinone glucosides. It is noted that the anti-inflammatory and anti-fungal potential of emodin (**9**) decreased when it become glycosides or seco-anthraquinone cleavaged between C-10 and C-4a.

## Experimental

### General experimental procedures

UV spectra were given on a UV-2410PC Shimadzu spectrometer. One and two-dimensional NMR spectra were determined on acetone-*d*_6_ and methanol-*d*_4_ with Bruker Ascend-600 and AV-800 spectrometers. Chemical shifts (*δ*) were recorded in (parts per million, ppm) scale with TMS (Bruker, Zurich, Switzerland) as an internal standard. Coupling constants were expressed in hertz (Hz). ESI mass spectra were measured on a VG Auto Spec300 spectrometer. High-resolution electro-spray ionization mass (HRESIMS) spectra were performed on an API QSTAR Pular-1 spectrometer. Semi-preparative HPLC was performed on a Hanbon Sci & Tech with Capcell Pak Phenyl (250 mm × 10 mm, 5 μm) and Thermo Hypeersil GOLD aQ (250 mm × 9.4 mm × 5 μm) columns. Analytical HPLC was performed on a Waters 2695 Series HPLC system equipped with a reverse-phase ZORBAX SB-C-18 column (4.6 mm × 150 mm, 5 μm, Agilent Corporation, USA). Column chromatography (CC) was carried out using Sephadex LH-20 (25–100 μm, Pharmacia Fine Chemical Co., Ltd., Uppsala, Sweden), 75–100 μm MCI-gel CHP20P (Mitsubishi Chemical Co. Ltd., Tokyo, Japan), silica gel (100–200 mesh, Qingdao Marine Chemical, Inc., Qingdao, China) and macro-porous absorption resin (D101, Donghong Chemical Co., Ltd., People’s Republic of China). Acetonitrile (chromatographic grade) were purchased from XinLanJing (Pennsylvania, USA). Mouse mononuclear macrophage RAW264.7 was purchased from the Shanghai Cell Bank of the Chinese Academy of Sciences, DMEM medium and fetal bovine serum were purchased from BI Company. Griess Reagent, LPS, Terbinafine hydrochloride, DMSO and control drug L-NMMA were purchased from Sigma. *Epidermophyton floccosum*, *Trichophyton rubrum* and *Microsporum gypseum* were purchased from the Medical Fungal Conservation Centre, Chinese Academy of Medical Sciences.

### Plant materials

The roots of *R. crispus* were collected from Yimen town, Xianyang City, Shaanxi Province, in August 2020, and identified by Dr. En-De Liu from Kunming Institute of Botany (KIB), Chinese Academy of Sciences (CAS). A voucher specimen (KIBZL-20200803) is deposited at State Key Laboratory of Phytochemistry and Plant Resource in West China of KIB–CAS.

### Extraction and isolation

The air-dried roots of *R. crispus* (10.0 kg) were crushed into small pieces and extracted with 90% aqueous MeOH at 60 °C (15 L × 4, each time 2 h). The organic solvent was removed under reduced pressure to yield a residue (1.6 kg), which was further extracted with ethyl acetate. After concentrated, the aqueous layer (480 g) was applied to a Sephadex LH-20 column chromatography (CC), eluting with water–methanol (1:0–0:1) to give two fractions (I–II). Fr. I (70 g) was subjected to CC over macroporous resin D101, eluting with H_2_O firstly to remove the sugars, and then with 100% MeOH. The yielded MeOH fraction (50.5 g) was subjected to CC over silica gel, eluting with a CHCl_3_/MeOH gradient system (1:0, 9:1, 8:2, 7:3, 6:4, 1:1, 0:1) to yield 6 fractions, A-F. Fr. A (10 g) was chromatographed on silica gel column with a petroleum ether/ethyl acetate gradient system (1:0, 9:1, 8:2, 7:3, 6:4, 1:1, 0:1) to yield **11** (4.8 mg), **10** (5.0 mg), **9** (100.0 mg). Fr. B (19.5 g) was appplied to RP-18 CC with a MeOH/H_2_O gradient system (from 0:1 to 1:0) to afford fractions B1-B4. Fr. B2 (500 mg) was purified by semipreparative HPLC (3 mL/min) with 7% MeCN/H_2_O (7:93) containing 0.1% trifluoroacetate to yield **1** (7.0 mg, retention time = 9 min), **6** (12.5 mg, retention time = 7 min), **7** (37.0 mg, retention time = 8 min), **8** (14.0 mg, retention time = 12 min), **12** (1.50 mg, retention time = 14 min), **13** (3.70 mg), **14** (17.0 mg). Fr. C (2.5 g) was separated by chromatography on a Chromatorex ODS column (2.5 cm i.d. × 35 cm) with 10 − 60% MeOH (5% stepwise, each 500 mL) to give Fr. C1 (50 mg) and Fr. C2 (1.0 g). Further, Fr. C1 was separated by semipreparative HPLC (Capcell Pak Phenyl, 250 mm × 10 mm × 5 μm, CH_3_CN/H_2_O 22:78) to obtain **2** (5.0 mg, retention time = 13.5 min) and **3** (12.0 mg, retention time = 17.3 min), and Fr. C2 was separated by semipreparative HPLC (Thermo Hypeersil GOLD aQ, 250 mm × 9.4 mm × 5 μm, CH_3_CN/H_2_O 28:72) to obtain **4** (50.0 mg, retention time = 8.0 min), **5** (305.0 mg, retention time = 10.0 min), respectively.

#### Crispuside A (1)

Yellowish amorphous powder; UV (MeOH) *λ*_max_ (log ε) 206 (4.4), 274 (3.7) nm. ^1^H (600 MHz) and ^13^C (150 MHz) NMR (in methanol-*d*_4_) data, see Table 1. ESIMS *m/z* 449 [M−H]^−^; HRESIMS *m/z* 449.1094 [M−H]^−^, (calcd C_21_H_21_O_11_: 449.1089).

#### Naphthalenone A (2)

White amorphous powder, ${\left[\alpha \right]}_{\mathrm{D}}^{19.5}$ + 15.03 (*c* 0.07, MeOH); UV (MeOH) *λ*_max_ (log ε) 222 (3.1) 270 (2.8), 330 (2.3) nm. ^1^H (600 MHz) and ^13^C (150 MHz) NMR (in methanol-*d*_4_) data, see Table 2. ESIMS *m/z* 235 [M−H]^−^; HRESIMS *m/z* 235.0979 [M−H]^−^, (calcd C_13_H_15_O_4_, 235.0976).

#### Naphthalenone B (3)

White amorphous powder; ${\left[\alpha \right]}_{\mathrm{D}}^{19.5}$ + 1.58 (*c* 0.16, MeOH); UV (MeOH) *λ*_max_ (log ε) 222 (3.1), 270 (2.8), 330 (2.3) nm. ^1^H (600 MHz) and ^13^C (150 MHz) NMR (in methanol-*d*_4_) data, see Table 2. ESIMS *m/z* 235 [M−H]^−^; HRESIMS *m/z* 235.0979 [M−H]^−^, (calcd C_13_H_15_O_4_, 235.0976).

#### Naphthalenone C (4)

Yellow amorphous powder; ${\left[\alpha \right]}_{\mathrm{D}}^{25.9}$ + 13.43 (*c* 0.76, MeOH); UV (MeOH) *λ*_max_ (log ε) 240 (3.1), 330 (2.6) nm. ^1^H (600 MHz) and ^13^C (150 MHz) NMR (in methanol-*d*_4_) data, see Table 2. ESIMS *m/z* 233 [M−H]^−^; HRESIMS *m/z* 233.0814 [M−H]^−^, (calcd C_13_H_13_O_4_ 233.0814).

#### (1*R*,2*R*,3*S*,4*S*)-3-Acetyl-2-methyl-1,4,5-trihydroxy-2,3-epoxynaphthoquinol (5)

White amorphous powder; ^1^H (500 MHz) and ^13^C (125 MHz) NMR (in methanol-*d*_4_) data, see Table 2. ESIMS *m/z* 249 [M−H]^−^.

#### Single-crystal X-ray diffraction data of 5

Colorless crystal of **5**: C_13_H_14_O_5_, *M* = 250.24, *a* = 7.7634 (3) Å, *b* = 8.2971 (3) Å, *c* = 9.2218(3) Å, *α* = 90°, *β* = 107.7570 (10)°, *γ* = 90°, *V* = 565.71 (4) Å^3^, *T* = 100 (2) K, space group *P*1211, *Z* = 2, *μ*(Cu Kα) = 0.954 mm^−1^, 10,180 reflections measured, 2156 independent reflections (*R*_*int*_ = 0.0500). The final *R*_*1*_ values were 0.0319 [*I* > 2*σ*(*I*)]. The final *wR*(*F*^2^) values were 0.0821 [*I* > 2*σ*(*I*)]. The final *R*_*1*_ values were 0.0333 (all data). The final *wR*(*F*^2^) values were 0.0832 (all data). The goodness of fit on *F*^2^ was 1.064. Flack parameter = 0.17(10). Crystallographic data for the structure of **5** have been deposited in the Cambridge Crystallographic Data Centre (deposition number CCDC, 2,158,643). Copies of the data can be obtained free of charge from the CCDC via www.ccdc.cam.ac.uk.

### The biological assay

The isolates **1** and **6**–**14** were evaluated for their anti-fungal against three skin fungi (*Epidermophyton floccosum*, *Trichophyton rubrum*, *Microsporum gypseum*) and anti-inflammatory activity. For anti-fungal activity, Terbinafine hydrochloride was used as positive control. Fungal broth (5 × 105 CFU mL^−1^) and test samples (100 μM) were incubated in 96-well plates at 25 °C for 5 days, A microplate reader was recorded by the absorbance at 625 nm. The experiment also set up the culture medium blank control, fungi control and terbinafine hydrochloride positive drug control.

The anti-inflammatory activity of **1** and **6**–**14** was screened as previously reported method [29]. The mouse mononuclear macrophage RAW264.7 was inoculated to 96 orifice plates and induced by 1.0 μg/mL LPS. At the same time, compounds **1** and **6**–**14** (final concentration 50 μM) was added, and no drug group and L-NMMA positive drug group were taken as controls. After the cells were cultured overnight, the medium was used to detect NO production and the absorbance was measured at 570 nm. MTS was added to the remaining medium to detect the cell survival rate and exclude the toxic effects of the compounds. The formula to calculate the inhibition rate is as follows: NO production inhibition rate (%) = (non-drug treatment group OD_570 nm_ − sample group OD_570 nm_)/non-drug treatment group OD_570 nm_ × 100%.

## Conclusion

Four new (**1**–**4**) and ten known (**5**–**14**) quinone derivatives were isolated and identified from the roots of *Rumex crispus* L. Compound **1** is a seco-anthraquinone glucoside, while **2**–**4** belong to naphthalenones containing 3,4-dihydronaphthalen-1(2*H*)-one moiety. The absolute configuration of **5** was determined for the first time by X-ray single crystal diffraction. The anti-fungal and anti-inflammatory activity of anthraquinones (**1**, **6**–**14**) was tested, of which compound **9** showed obvious anti-fungal activity. The results indicated that simple emodin type anthraquinone is more potential against skin fungi than its oxyglucosyl, *C*-glucosyl and glycosylated seco analogues.

## Supplementary Information

**Additional file 1.** Supporting information.
