# Supplementary material for: New seco-anthraquinone glucoside from the roots of Rumex crispus
Source: Nat Prod Bioprospect. 2022 Aug 3;12(1):29. doi: 10.1007/s13659-022-00350-3 (PMC9346041; doi:10.1007/s13659-022-00350-3)
Supplement: Supplementary file 1 — Additional file 1. Supporting information. [file 13659_2022_350_MOESM1_ESM.docx]

**Supporting Information**

**New seco-anthraquinone glucoside from the roots of *Rumex crispus***

Yong-Xiang Li^1,2^, Na Li^1^, Jing-Juan Li^1^, Man Zhang^1^, Hong-Tao Zhu^1^, Dong Wang^1^, Ying-Jun Zhang^1,3, *^

**Affiliations**

^1^ State Key Laboratory of Phytochemistry and Plant Resources in West China, Kunming Institute of Botany, Chinese Academy of Sciences, Kunming 650204, People’s Republic of China

^2^ University of Chinese Academy of Sciences, Beijing 100049, People’s Republic of China

^3^ Yunnan Key Laboratory of Natural Medicinal Chemistry, Kunming Institute of Botany, Chinese Academy of Sciences, Kunming 650201, People’s Republic of China

**Correspondence**

**Prof. Dr. Ying-Jun Zhang**

State Key Laboratory of Phytochemistry and Plant Resources in West China

Kunming Institute of Botany, Chinese Academy of Sciences

Kunming 650201

P. R. China

Tel/ Fax: +86-871-65223235

E-mail: [zhangyj@mail.kib.ac.cn](mailto:zhangyj@mail.kib.ac.cn)

**Table of contents**

**Contents Page**

[**Fig. S1** ^1^H NMR spectrum of compound **1** in CD_3_OD 5](#_Toc102033075)

[**Fig. S2** ^13^C and DEPT NMR spectra of compound **1** in CD_3_OD 6](#_Toc102033076)

[**Fig. S3** ^1^H-^1^H COSY spectrum of compound **1** in CD_3_OD 7](#_Toc102033077)

[**Fig. S4** HMBC spectrum of compound **1** in CD_3_OD 8](#_Toc102033078)

[**Fig. S5** HSQC spectrum of compound **1** in CD_3_OD 9](#_Toc102033079)

[**Fig. S6** ROESY spectrum of compound **1** in CD_3_OD 10](#_Toc102033080)

[**Fig. S7** The (-)-HRESIMS spectroscopic data of compound **1** 11](#_Toc102033081)

[**Fig. S8** The UV spectrum of compound **1** in CD_3_OD 12](#_Toc102033082)

[**Fig. S9** The ^1^H NMR spectrum of compound **2** in CD_3_OD 13](#_Toc102033083)

[**Fig. S10** The ^13^C and DEPT NMR spectra of compound **2** in CD_3_OD 14](#_Toc102033084)

[**Fig. S11** ^1^H-^1^H COSY spectrum of compound **2** in CD_3_OD 15](#_Toc102033085)

[**Fig. S12** HMBC spectrum of compound **2** in CD_3_OD 16](#_Toc102033086)

[**Fig. S13** HSQC spectrum of compound **2** in CD_3_OD 17](#_Toc102033087)

[**Fig. S14** ROESY spectrum of compound **2** in CD_3_OD 18](#_Toc102033088)

[**Fig. S15** The (-)-HRESIMS spectroscopic data of compound **2** 19](#_Toc102033089)

[**Fig. S16** The UV spectrum of compound **2** in CD_3_OD 20](#_Toc102033090)

[**Fig. S17** Experimental ECD spectrum of compound **2** in CD_3_OD 21](#_Toc102033091)

[**Fig. S18** OR of compound **2** in MeOH 22](#_Toc102033092)

[**Fig. S19** ^1^H NMR spectrum of compound **3** in CD_3_OD 23](#_Toc102033094)

[**Fig. S20** ^13^C and DEPT NMR spectra of compound **3** in CD_3_OD 24](#_Toc102033095)

[**Fig. S21** ^1^H-^1^H COSY spectrum of compound **3** in CD_3_OD 25](#_Toc102033096)

[**Fig. S22** HMBC spectrum of compound **3** in CD_3_OD 26](#_Toc102033097)

[**Fig. S23** HSQC spectrum of compound **3** in CD_3_OD 27](#_Toc102033098)

[**Fig. S24** ROESY spectrum of compound **3** in CD_3_OD 28](#_Toc102033099)

[**Fig. S25** The (-)-HRESIMS spectroscopic data of compound **3** 29](#_Toc102033100)

[**Fig. S26** The UV spectrum of compound **3** in CD_3_OD 30](#_Toc102033101)

[**Fig. S27** Experimental ECD spectrum of compound **3** in CD_3_OD 31](#_Toc102033102)

[**Fig. S28** OR of compound **3** in MeOH 32](#_Toc102033103)

[**Fig. S29** The ^1^H NMR spectrum of compound **4** in CD_3_OD 33](#_Toc102033104)

[**Fig. S30** The ^13^C and DEPT NMR spectra of compound **4** in CD_3_OD 34](#_Toc102033105)

[**Fig. S31** ^1^H-^1^H COSY spectrum of compound **4** in CD_3_OD 35](#_Toc102033106)

[**Fig. S32** HMBC spectrum of compound **4** in CD_3_OD 36](#_Toc102033107)

[**Fig. S33** HSQC spectrum of compound **4** in CD_3_OD 37](#_Toc102033108)

[**Fig. S34** ROESY spectrum of compound **4** in CD_3_OD 38](#_Toc102033109)

[**Fig. S35** The (-)-HRESIMS spectroscopic data of compound **4** 39](#_Toc102033110)

[**Fig. S36** The UV spectrum of compound **4** in CD_3_OD 40](#_Toc102033111)

[**Fig. S37** Experimental ECD spectrum of compound **4** in CD3OD 41](#_Toc102033112)

[**Fig. S38** OR of compound **4** in MeOH 42](#_Toc102033113)

[**Fig. S39** The ^1^H NMR spectrum of compound **5** in CD_3_OD 43](#_Toc102033114)

[**Fig. S40** The ^13^C and DEPT NMR spectra of compound **5** in CD_3_OD 44](#_Toc102033116)

[**Fig. S41** ^1^H-^1^H COSY spectrum of compound **5** in CD_3_OD 45](#_Toc102033117)

[**Fig. S42** HMBC spectrum of compound **5** in CD_3_OD 46](#_Toc102033118)

[**Fig. S43** HSQC spectrum of compound **5** in CD_3_OD 47](#_Toc102033119)

[**Fig. S44** ROESY spectrum of compound **5** in CD_3_OD 48](#_Toc102033120)

[**Fig. S45** The (-)-ESIMS spectroscopic data of compound **5** 49](#_Toc102033121)

[**Table S1** Inhibitory effects of **1**, **6**-**14** against three strains skin fungi 50](#_Toc102033122)

[**Table S2** Anti-inflammatory effects of **1**, **6**-**14** 51](#_Toc102033123)


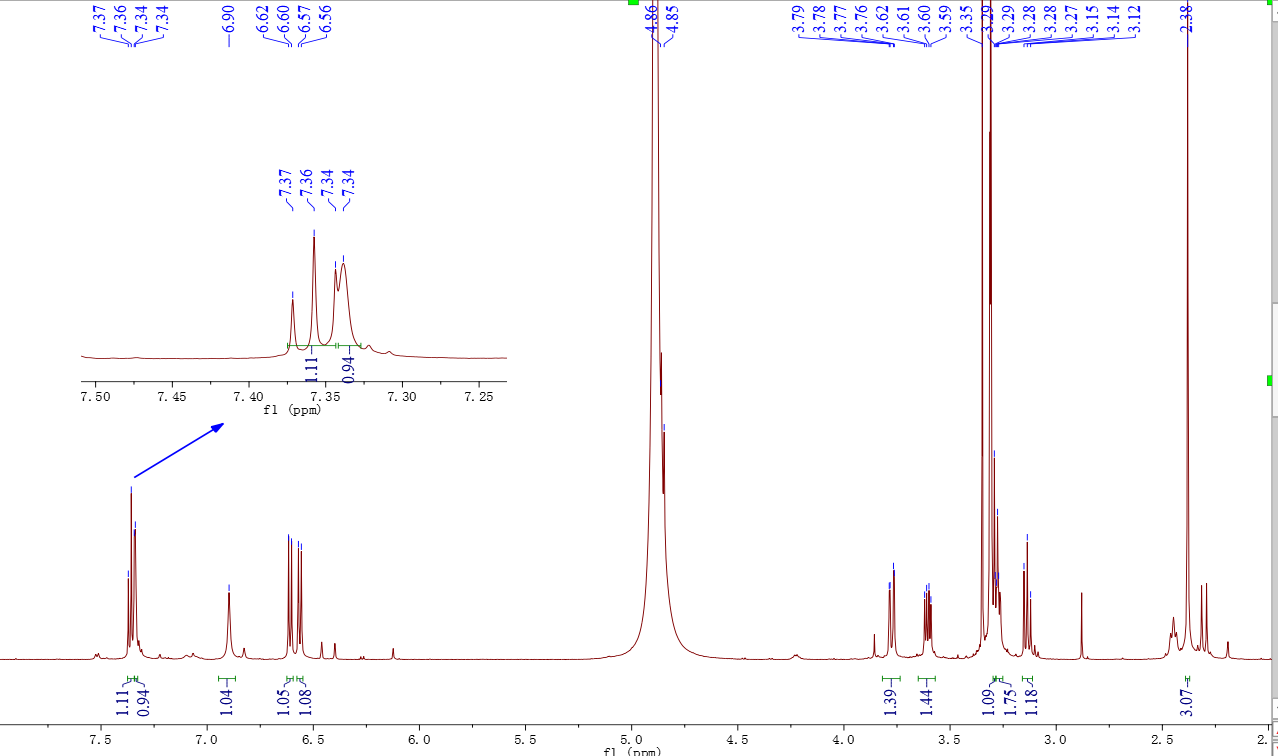


## **[Fig. S1](#_Toc61201451)** ^[1](#_Toc61201451)^[H NMR spectrum of compound](#_Toc61201451) **[1](#_Toc61201451)** [in CD](#_Toc61201451)_[3](#_Toc61201451)_[OD](#_Toc61201451)


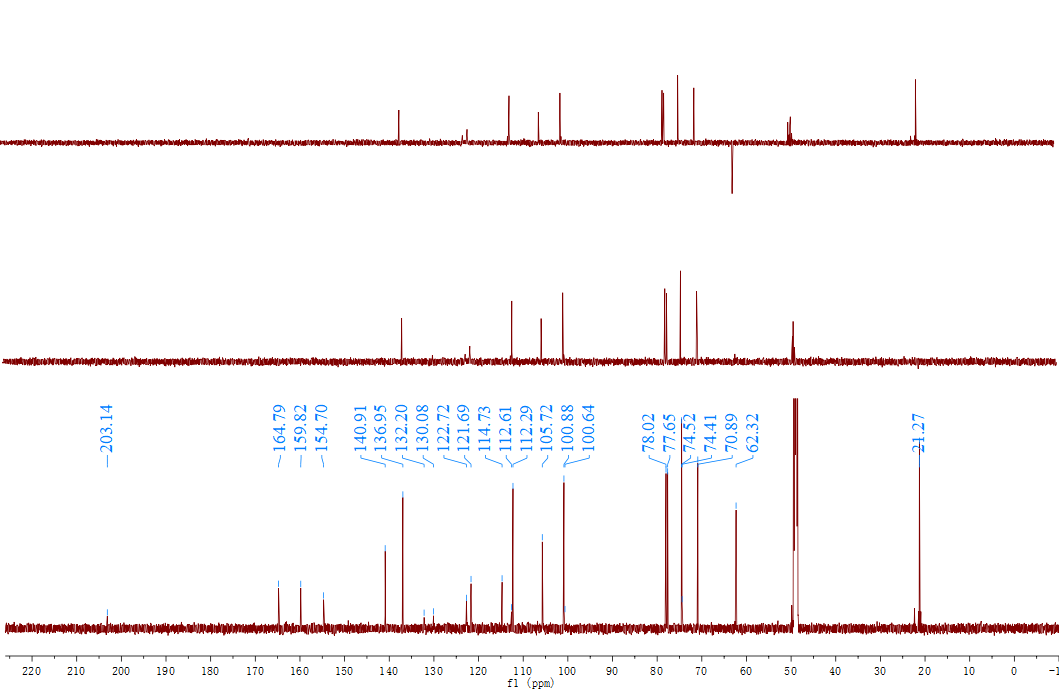


## [**Fig. S2** ^13^C and DEPT NMR spectra of compound **1** in CD_3_OD](#_Toc61201452)


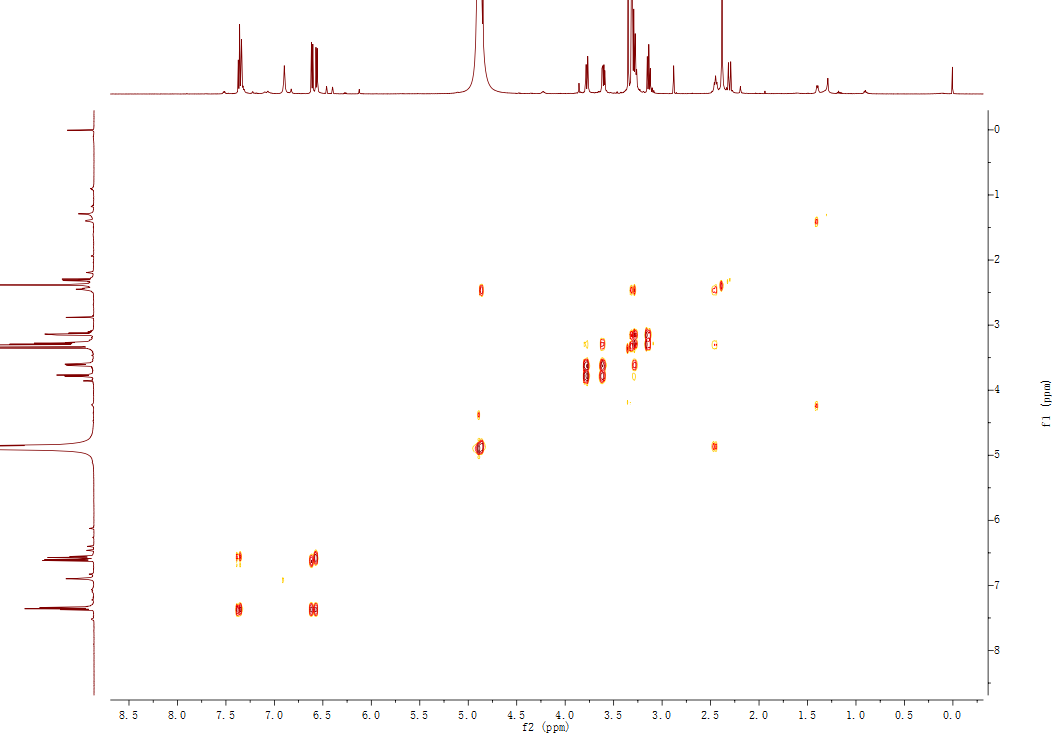


## [**Fig. S3** ^1^H-^1^H COSY spectrum of compound **1** in CD_3_OD](#_Toc61201455)


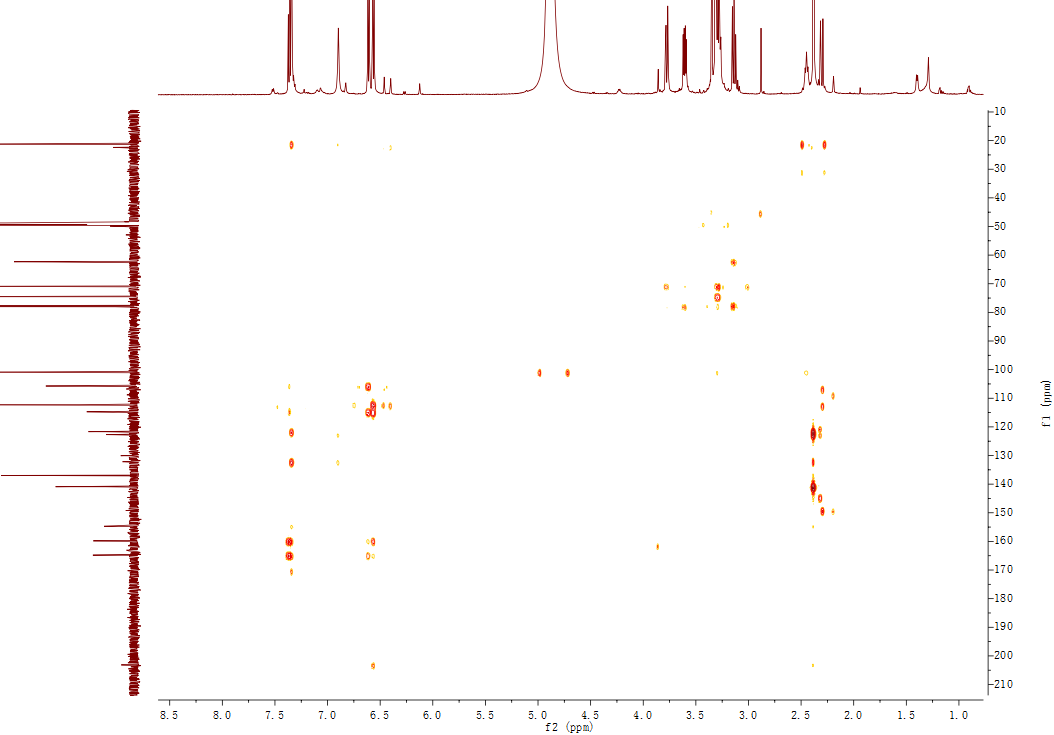


## [**Fig. S4** HMBC spectrum of compound **1** in CD_3_OD](#_Toc61201456)


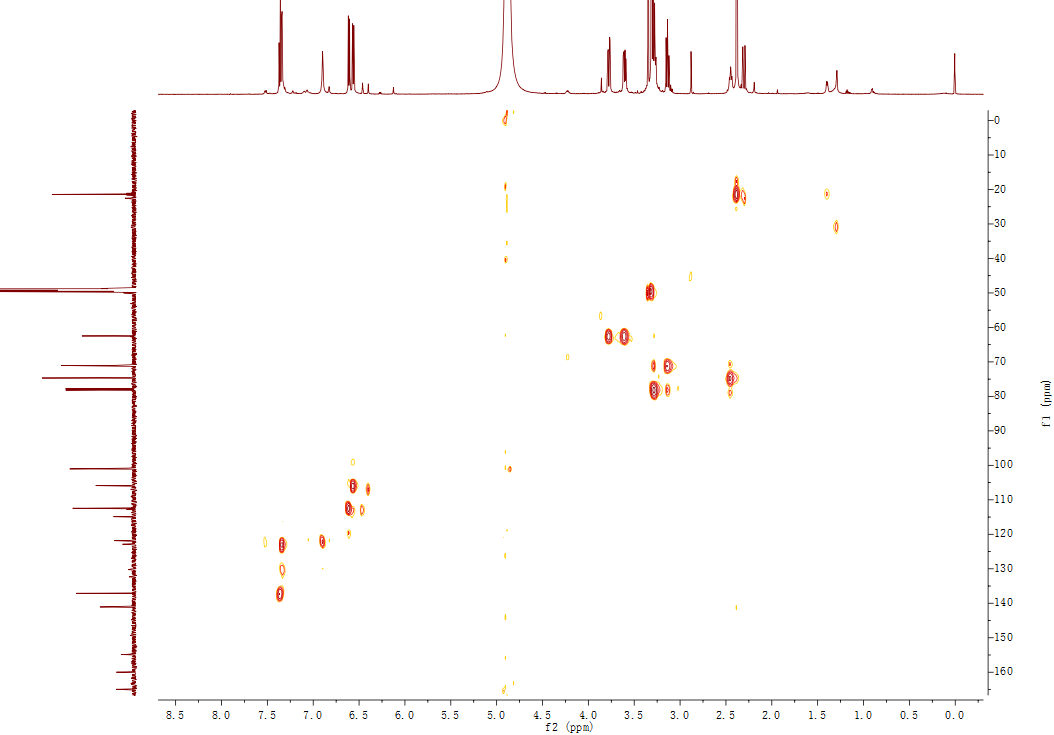


## [**Fig. S5** HSQC spectrum of compound **1** in CD_3_OD](#_Toc61201454)


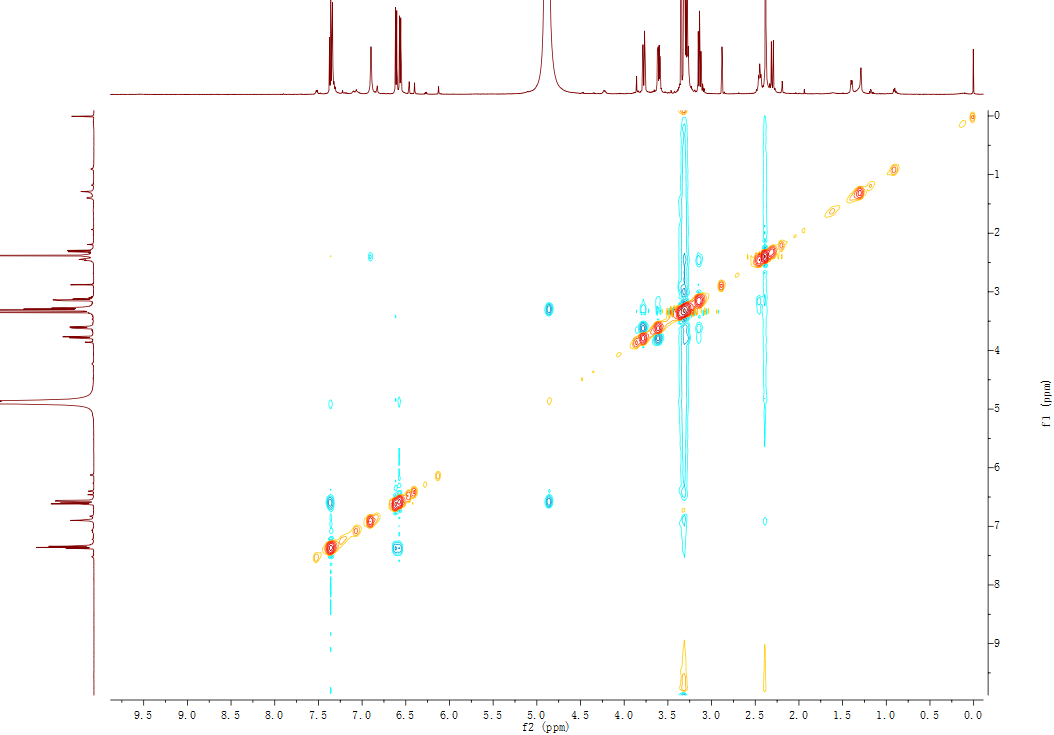


## [**Fig. S6** ROESY spectrum of compound **1** in CD_3_OD](#_Toc61201454)


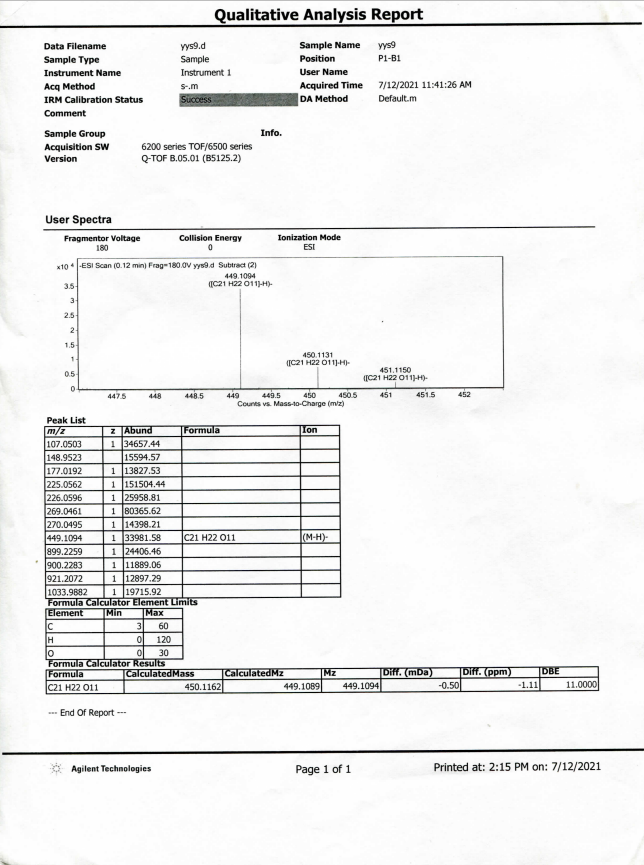


## [Fig. S7 The (-)-HRESIMS spectroscopic data of compound 1](#_Toc61201450)


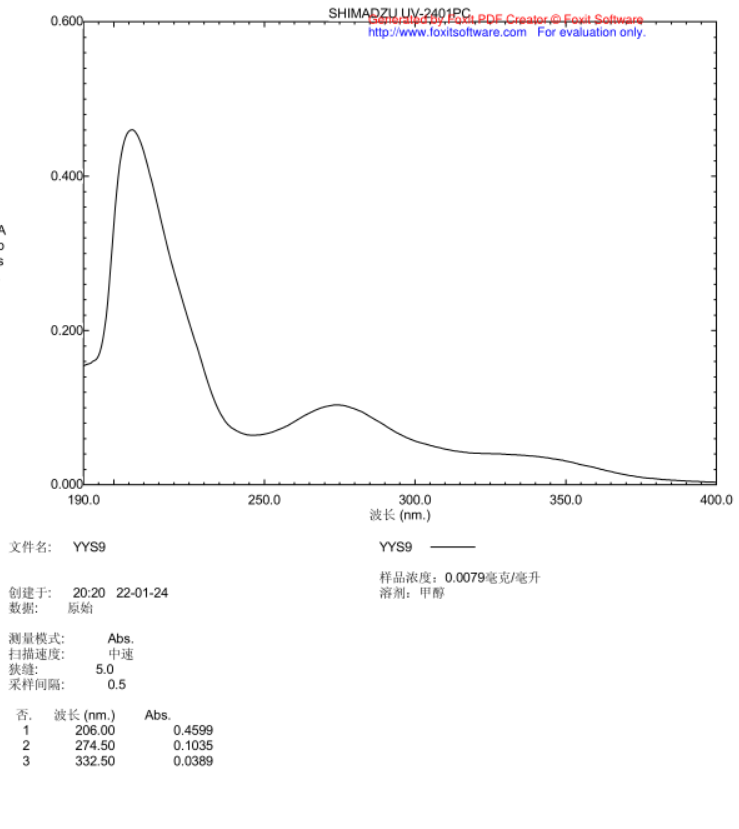


## **Fig. S8** The UV spectrum of compound **1** in CD_3_OD


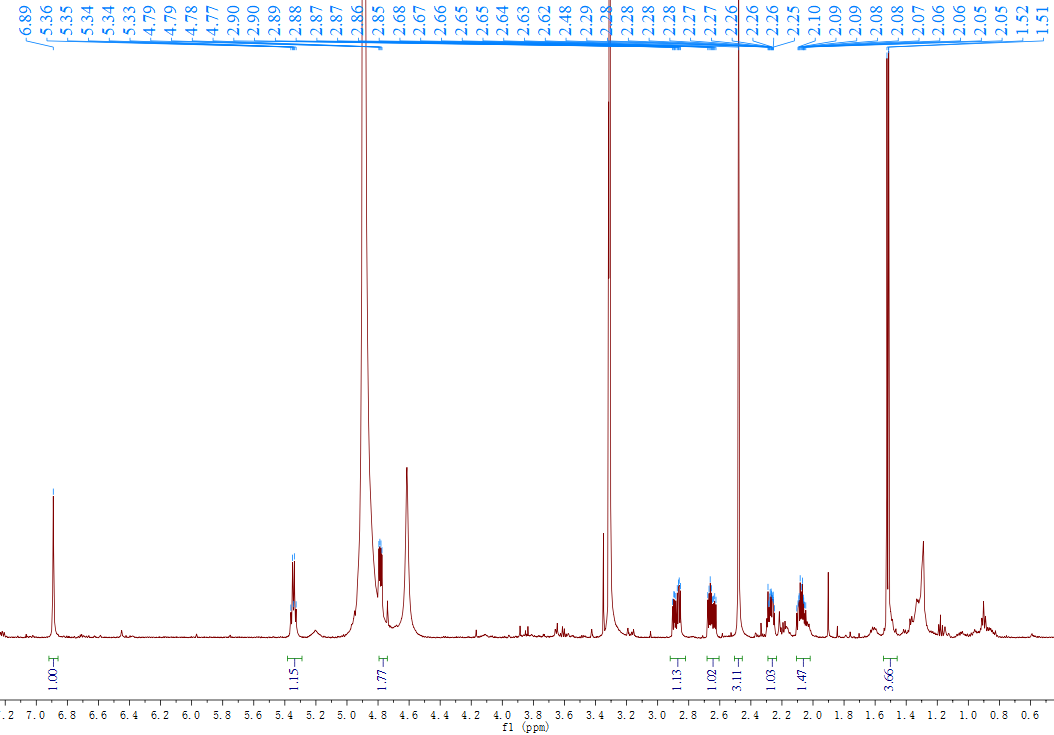


## **Fig. S9** The ^1^H NMR spectrum of compound **2** in CD_3_OD


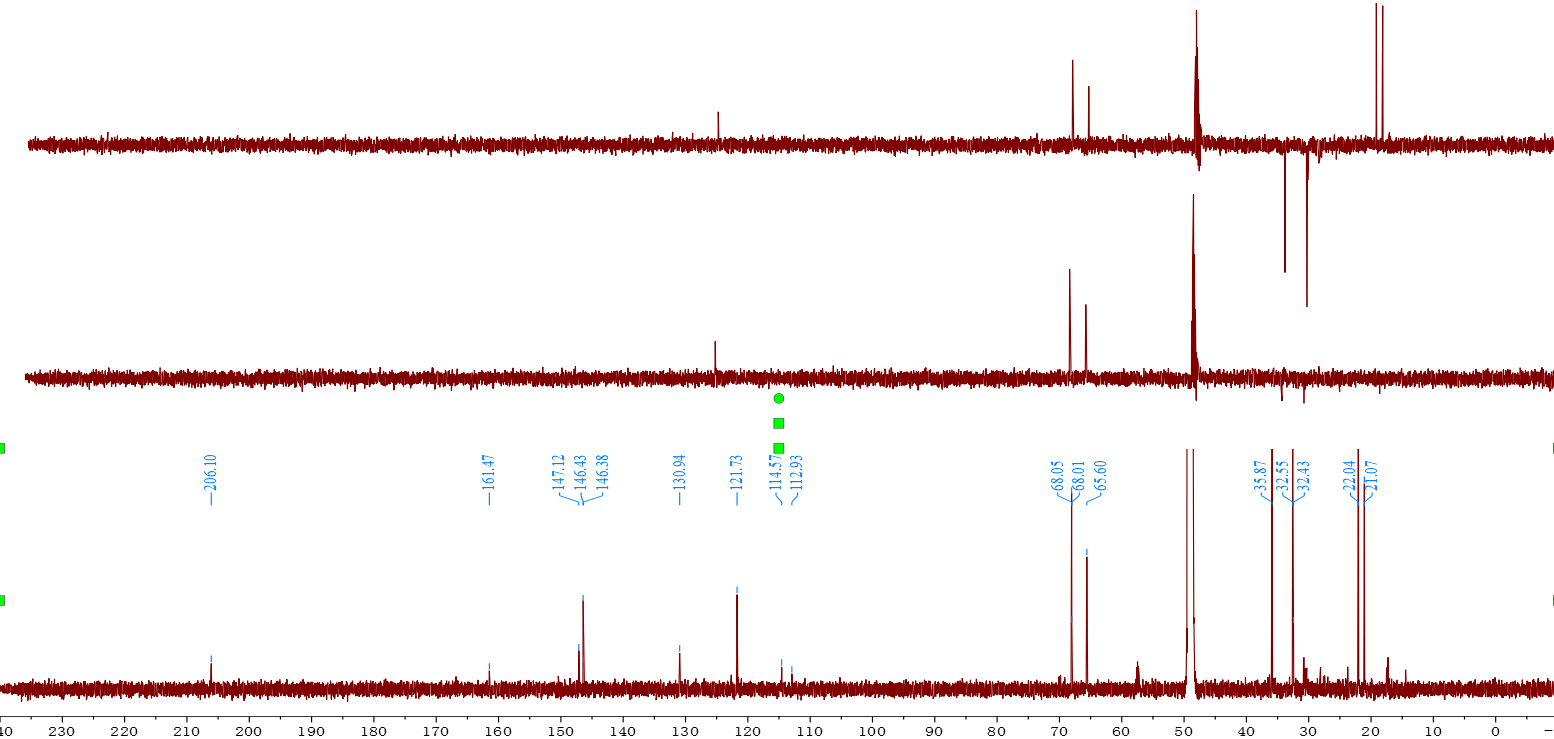


## **Fig. S10** The ^13^C and DEPT NMR spectra of compound **2** in CD_3_OD


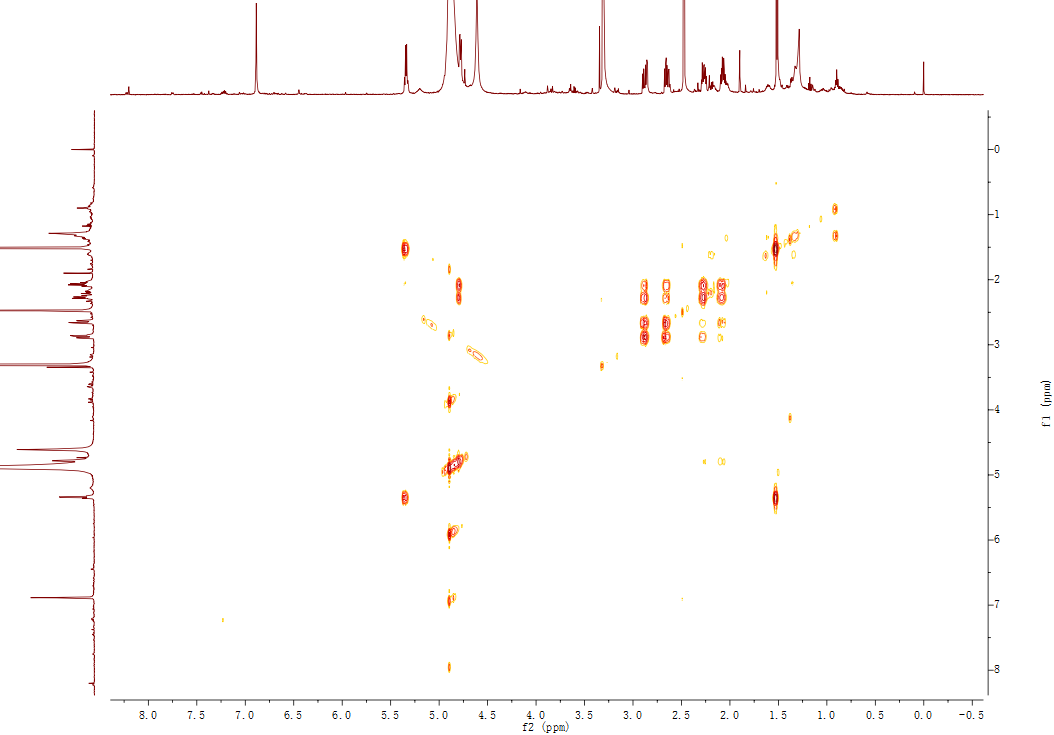


## **Fig. S11** ^1^H-^1^H COSY spectrum of compound **2** in CD_3_OD


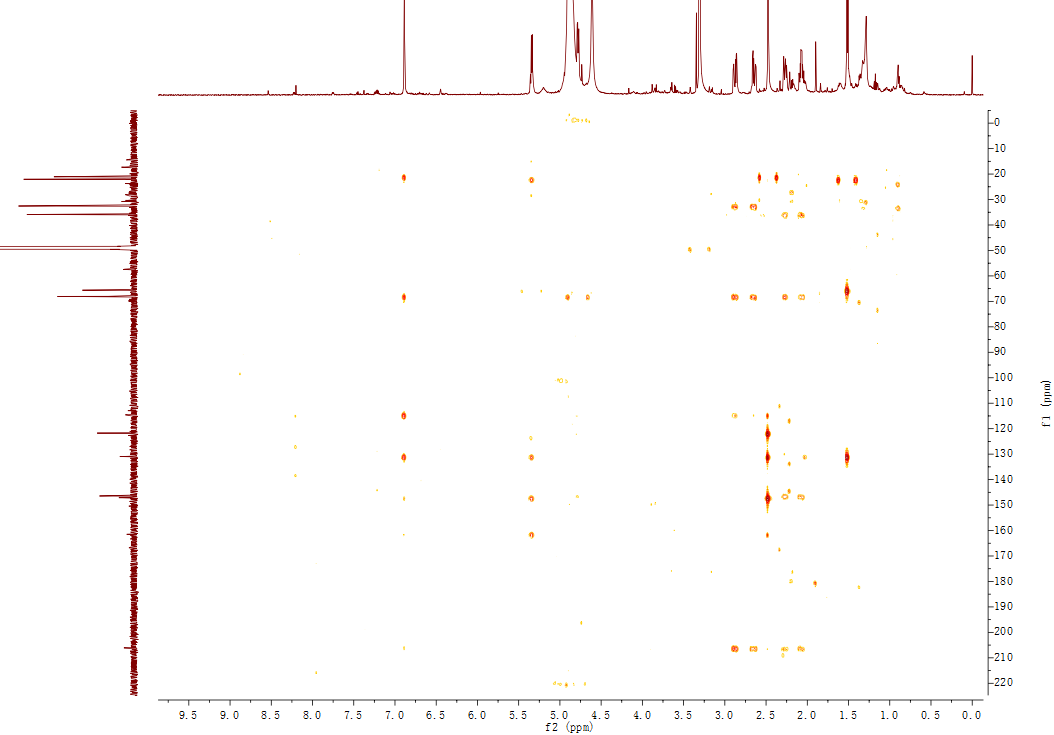


## **Fig. S12** HMBC spectrum of compound **2** in CD_3_OD


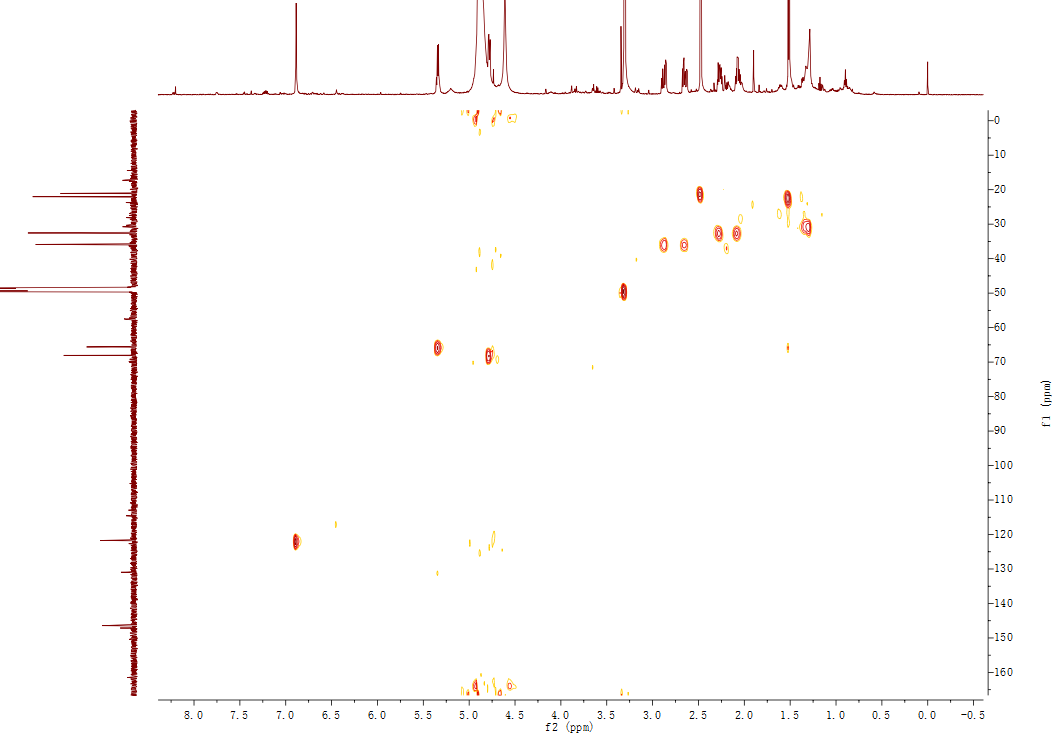


## **Fig. S13** HSQC spectrum of compound **2** in CD_3_OD


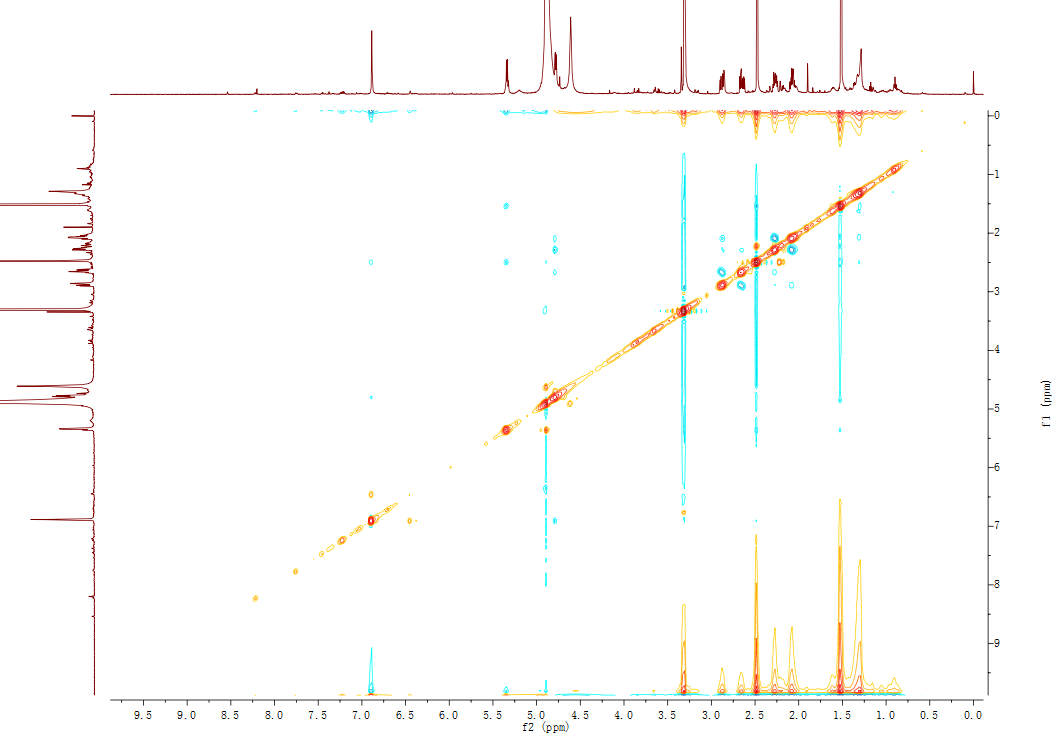


## [**Fig. S14** ROESY spectrum of compound **2** in CD_3_OD](#_Toc61201454)


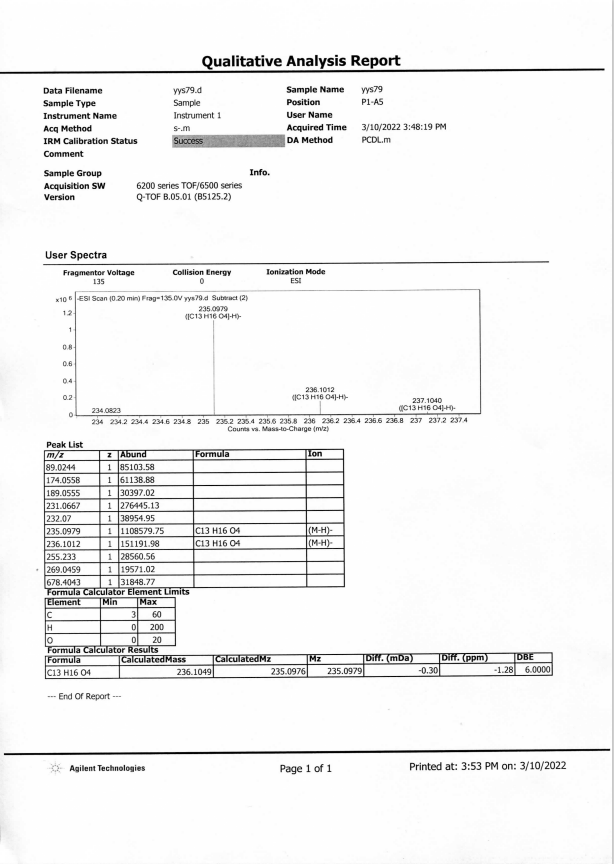


## [[**Fig. S15** The (-)-HRESIMS spectroscopic data of compound **2**](#_Toc61201450)](#_Toc61201456)


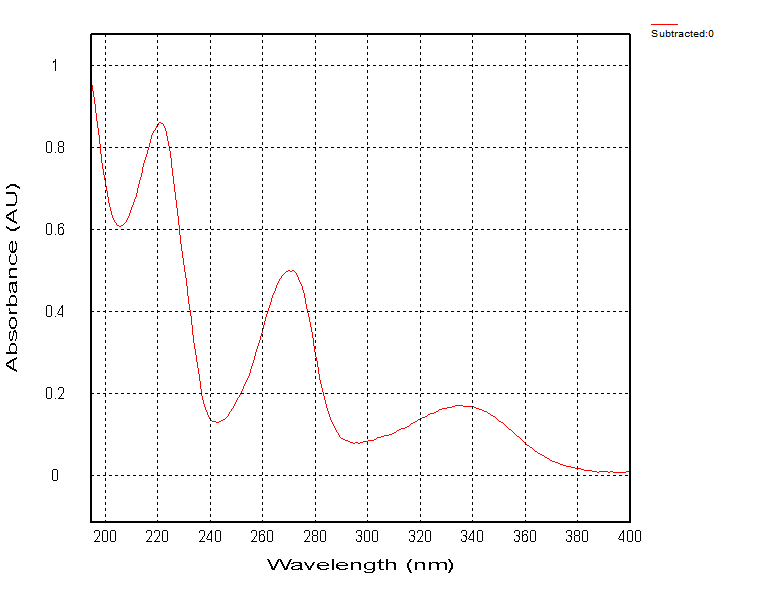


## **Fig. S16** The UV spectrum of compound **2** in CD_3_OD


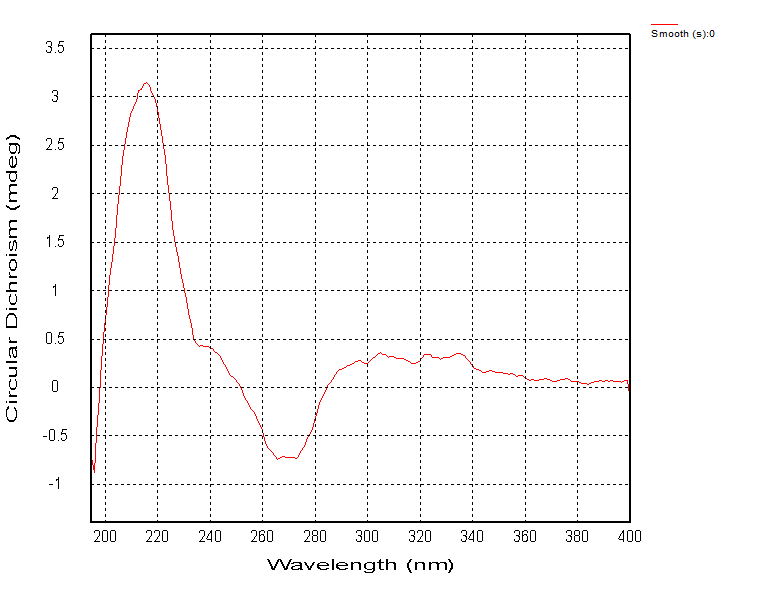


## **Fig. S17** Experimental ECD spectrum of compound **2** in CD_3_OD


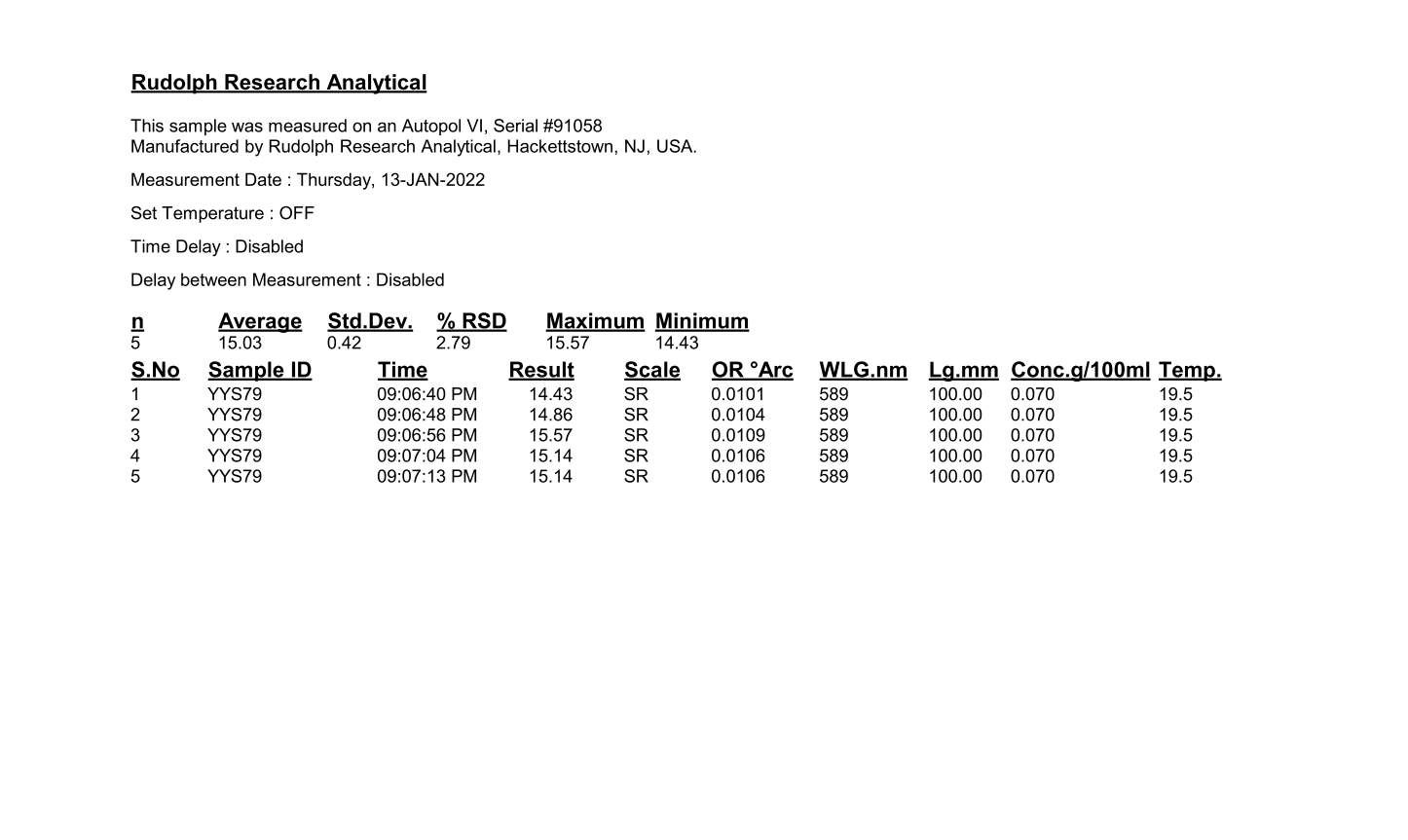


## **Fig. S18** OR of compound **2** in MeOH

##
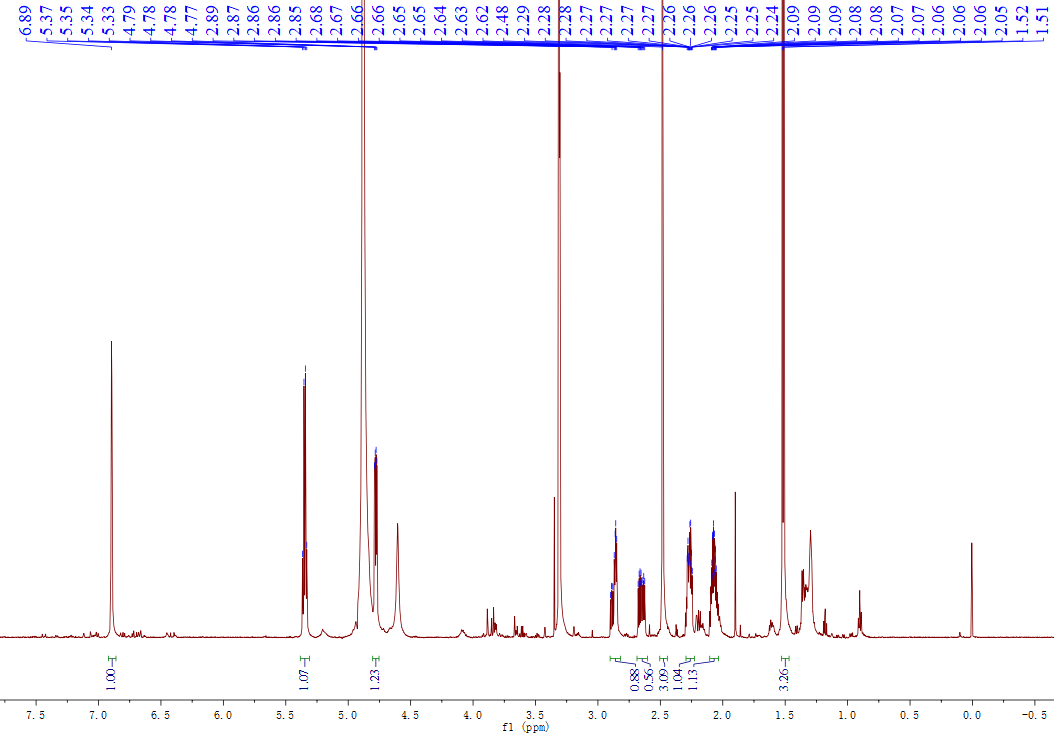


## [**Fig. S19** ^1^H NMR spectrum of compound **3** in CD_3_OD](#_Toc61201451)


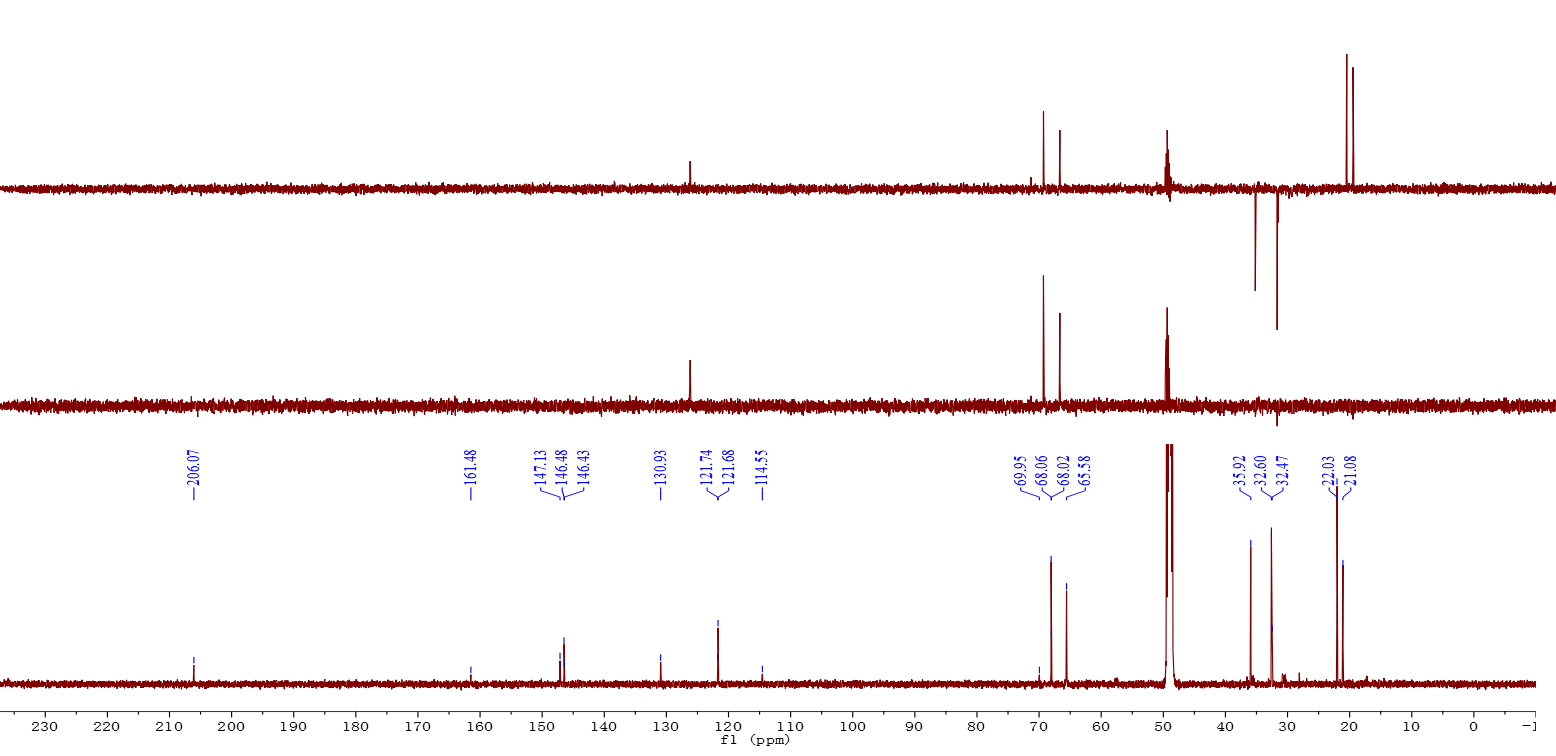


## [**Fig. S20** ^13^C and DEPT NMR spectra of compound **3** in CD_3_OD](#_Toc61201452)


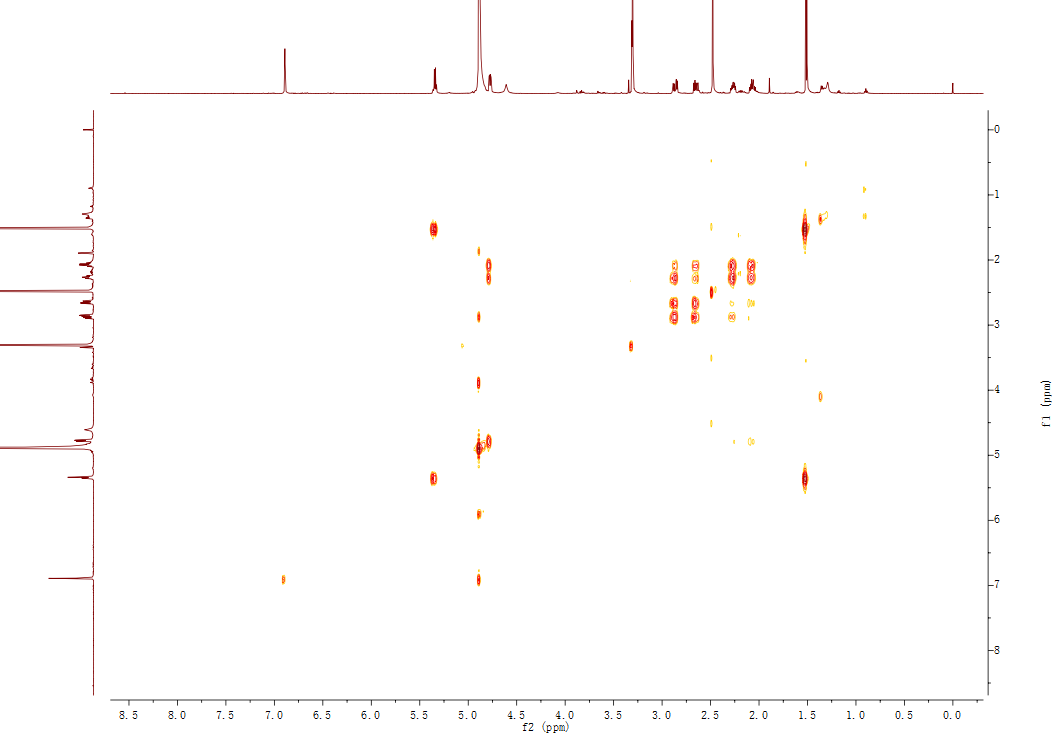


## [**Fig. S21** ^1^H-^1^H COSY spectrum of compound **3** in CD_3_OD](#_Toc61201455)


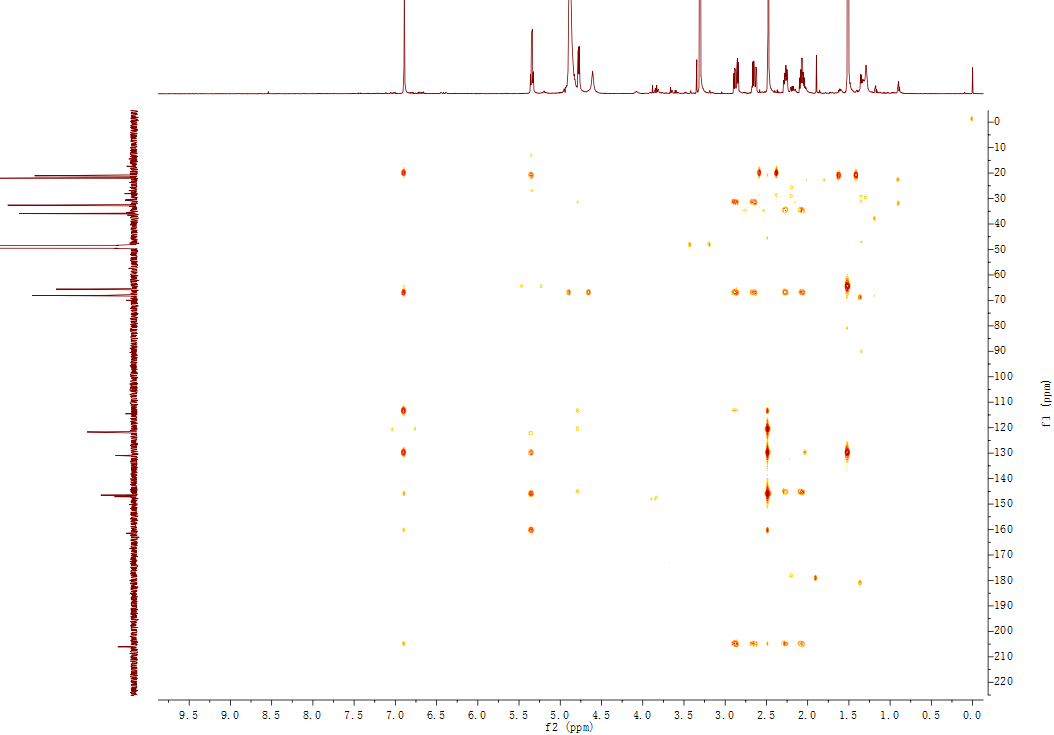


## [**Fig. S22** HMBC spectrum of compound **3** in CD_3_OD](#_Toc61201456)


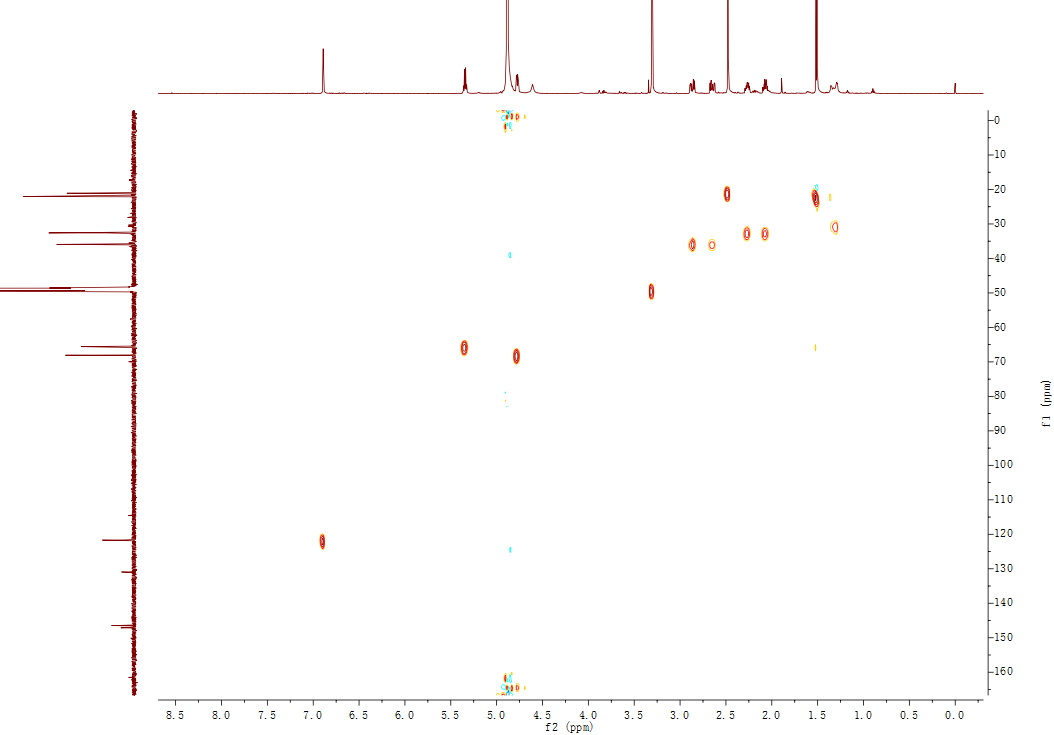


## [**Fig. S23** HSQC spectrum of compound **3** in CD_3_OD](#_Toc61201454)


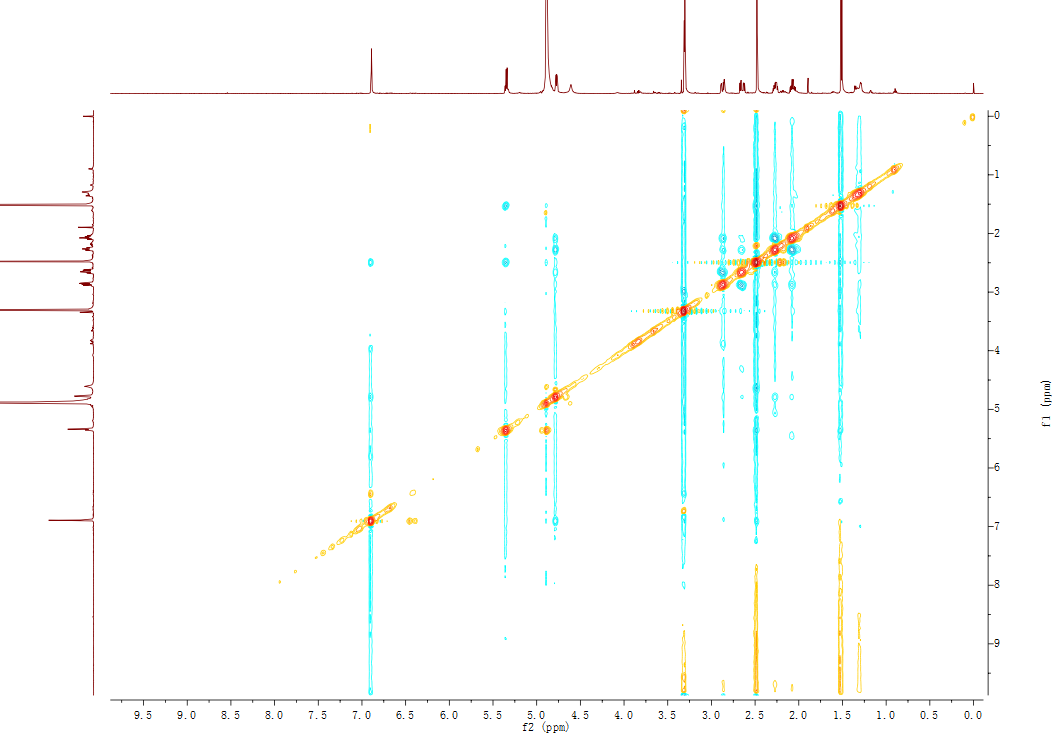


## [**Fig. S24** ROESY spectrum of compound **3** in CD_3_OD](#_Toc61201454)


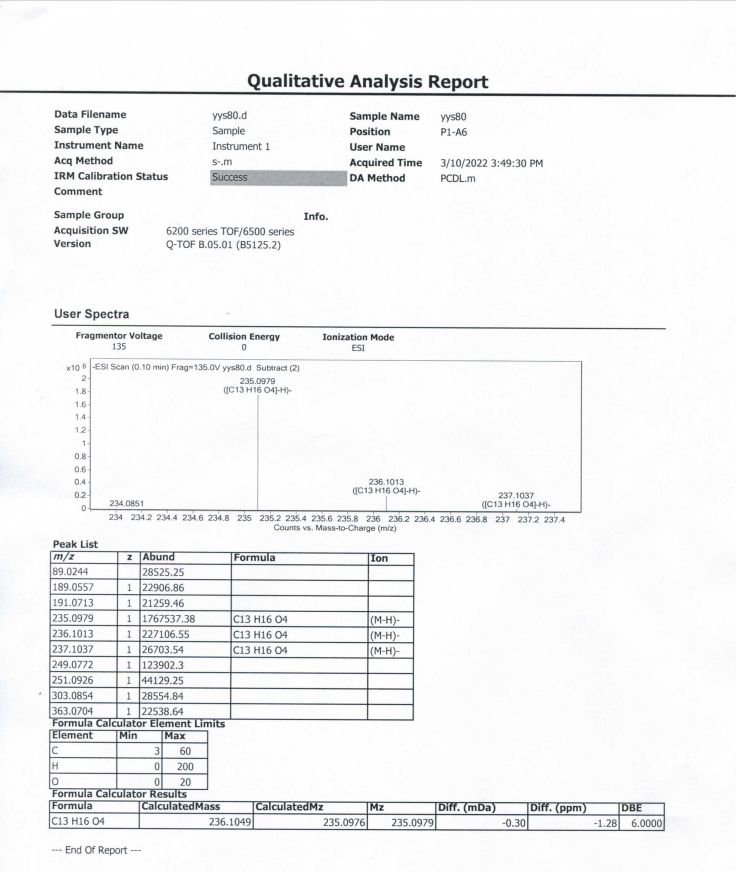


## [Fig. S25 The (-)-HRESIMS spectroscopic data of compound 3](#_Toc61201450)


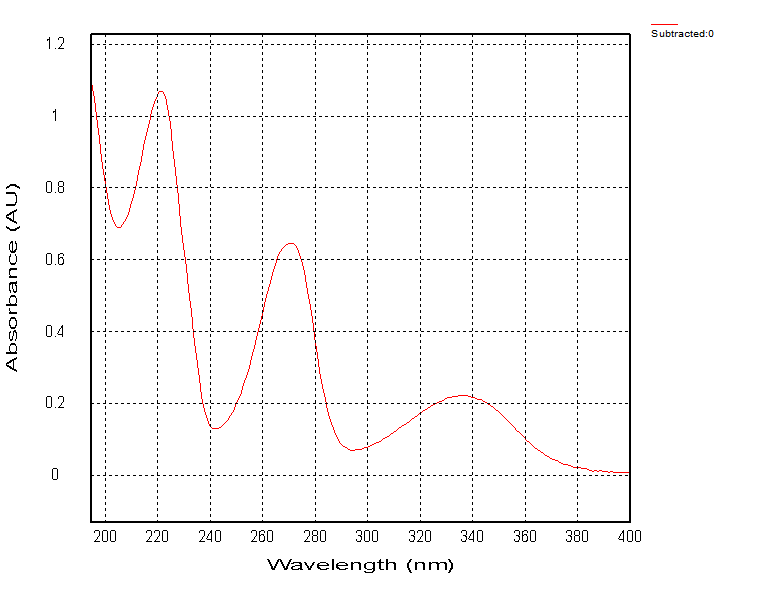


## **Fig. S26** The UV spectrum of compound **3** in CD_3_OD


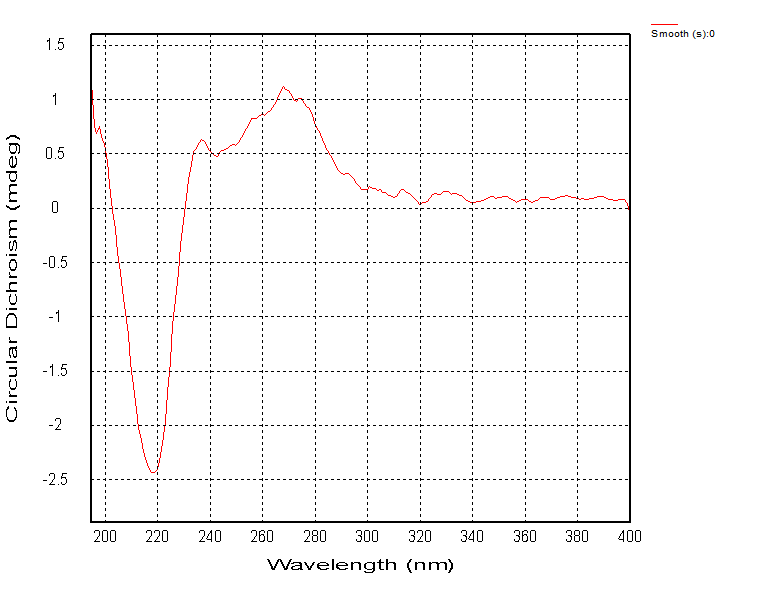


## **Fig. S27** Experimental ECD spectrum of compound **3** in CD_3_OD


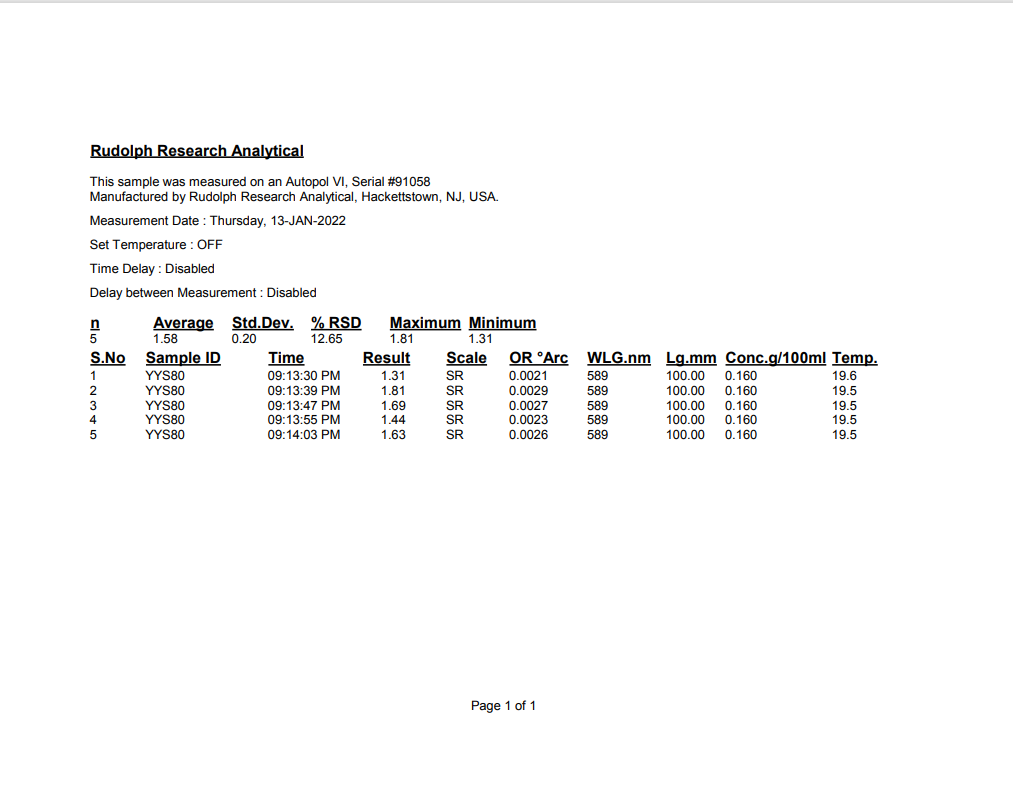


## **Fig. S28** OR of compound **3** in MeOH


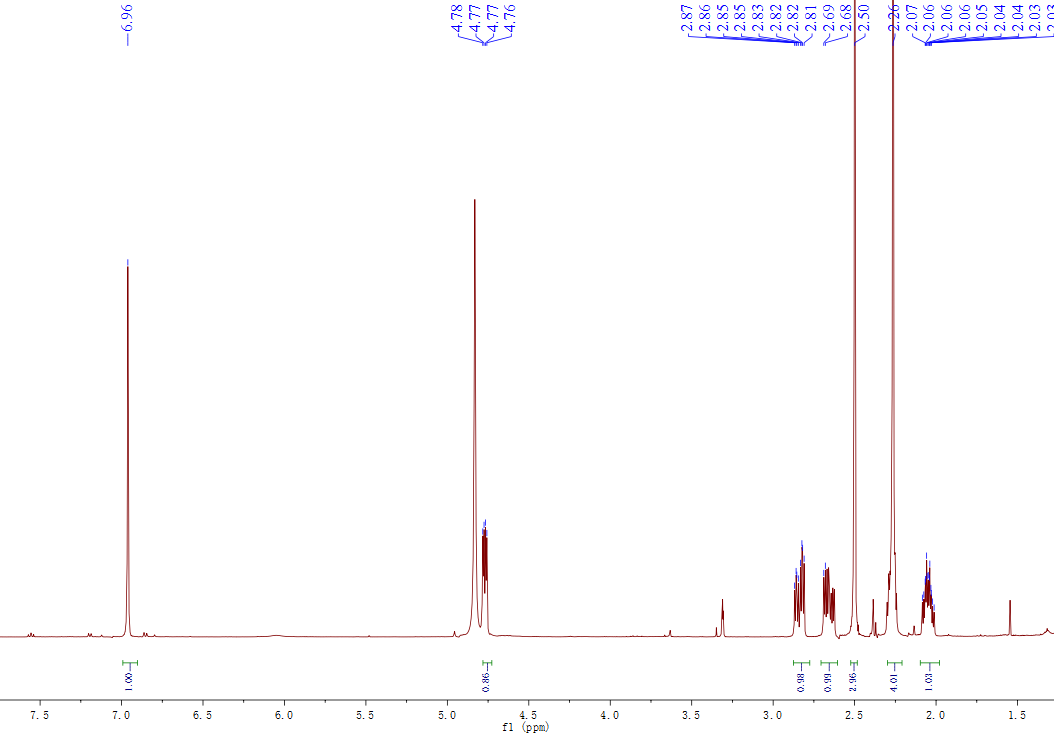


## **Fig. S29** The ^1^H NMR spectrum of compound **4** in CD_3_OD


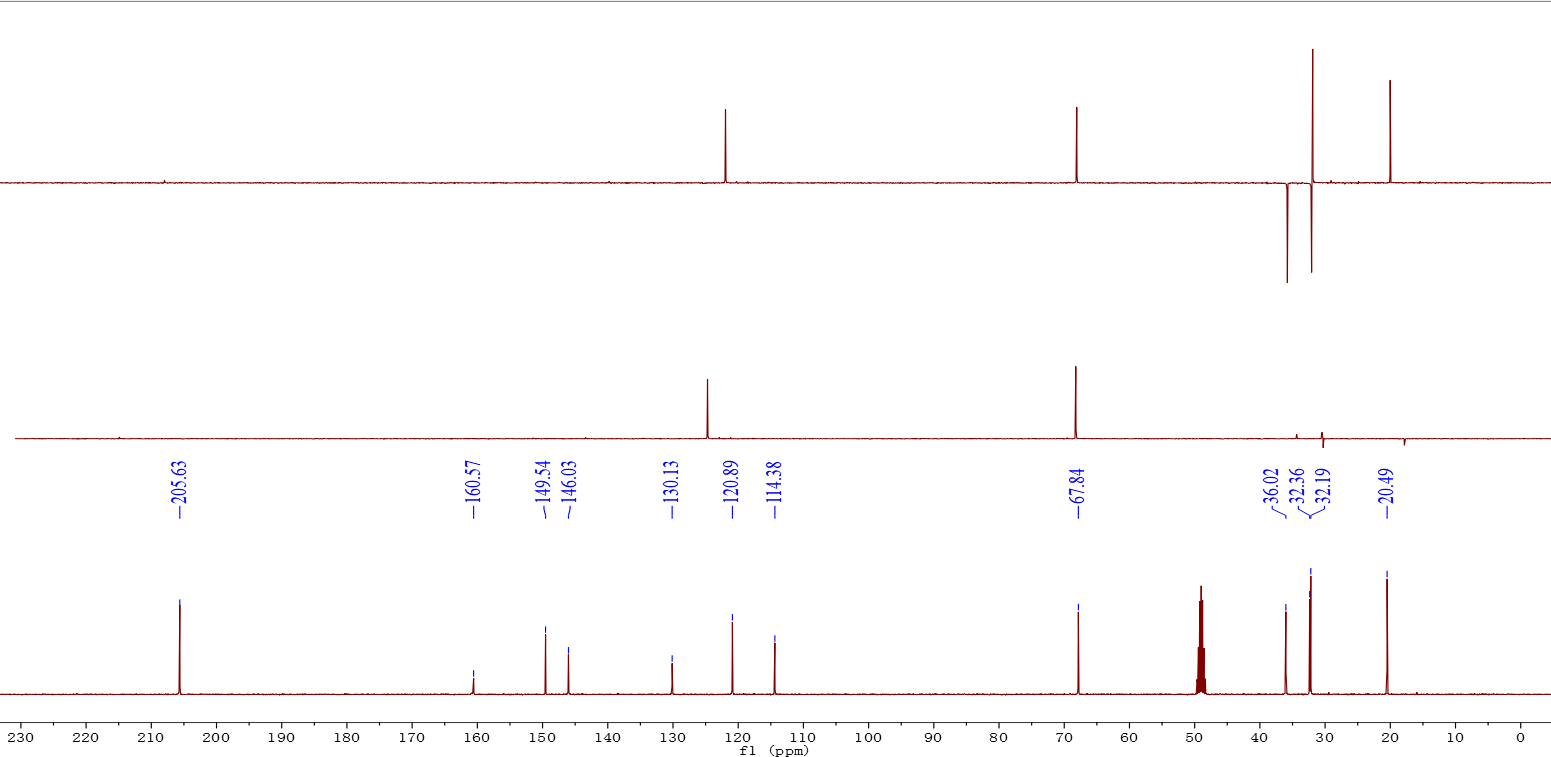


## **Fig. S30** The ^13^C and DEPT NMR spectra of compound **4** in CD_3_OD


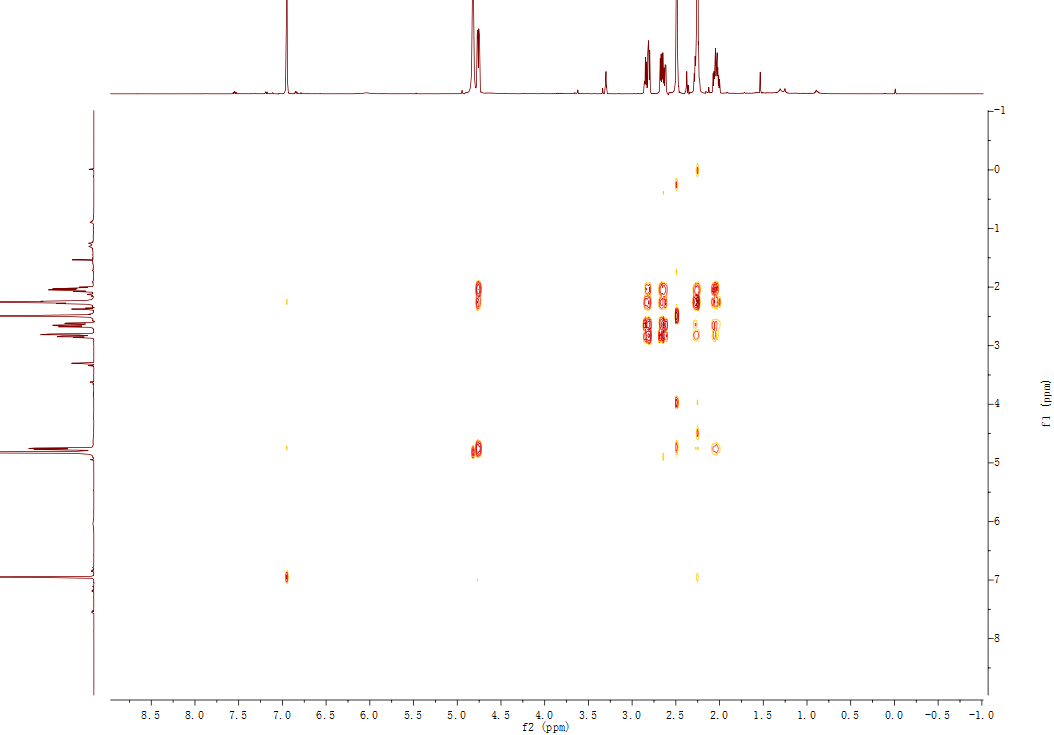


## **Fig. S31** ^1^H-^1^H COSY spectrum of compound **4** in CD_3_OD


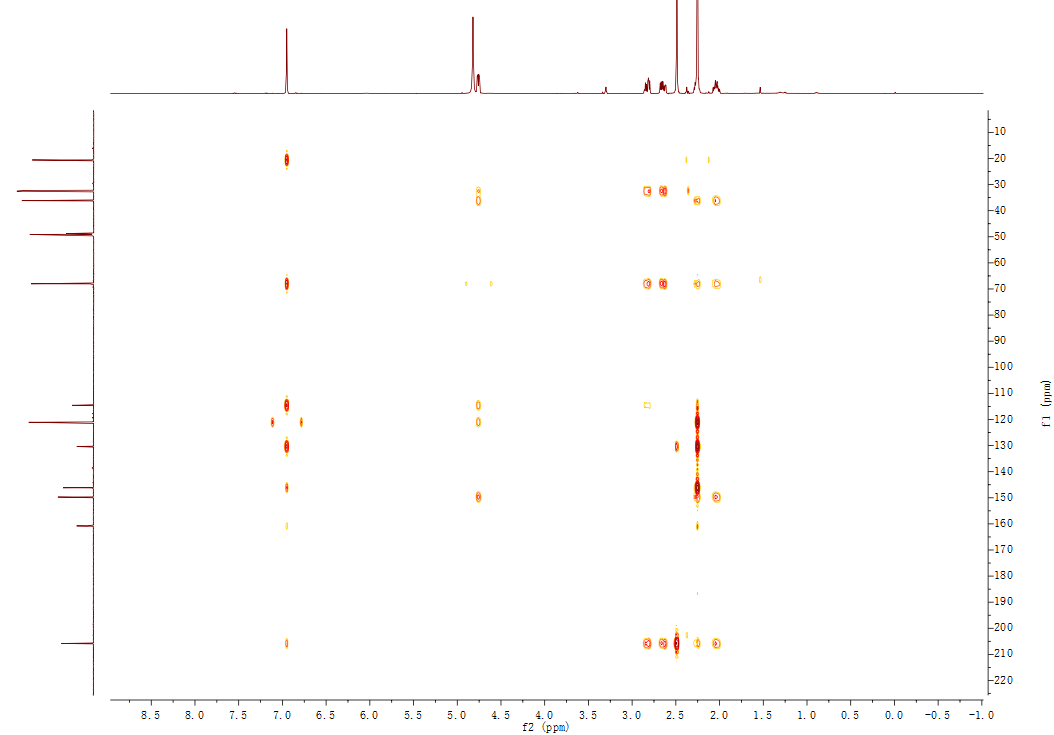


## **Fig. S32** HMBC spectrum of compound **4** in CD_3_OD


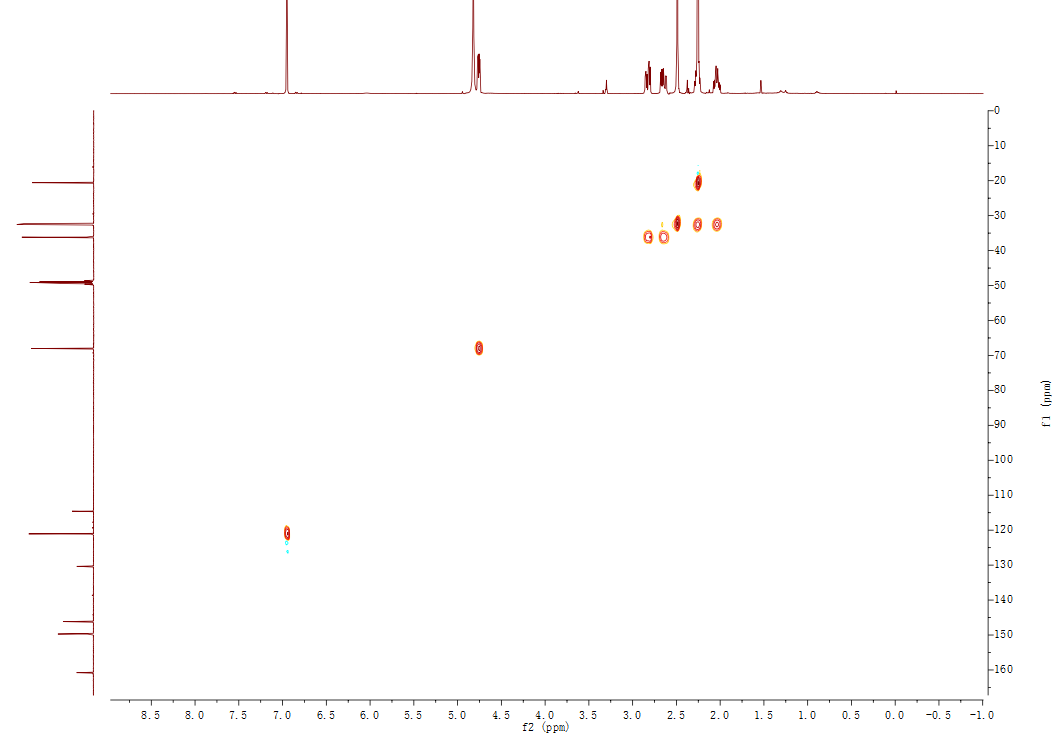


## **Fig. S33** HSQC spectrum of compound **4** in CD_3_OD


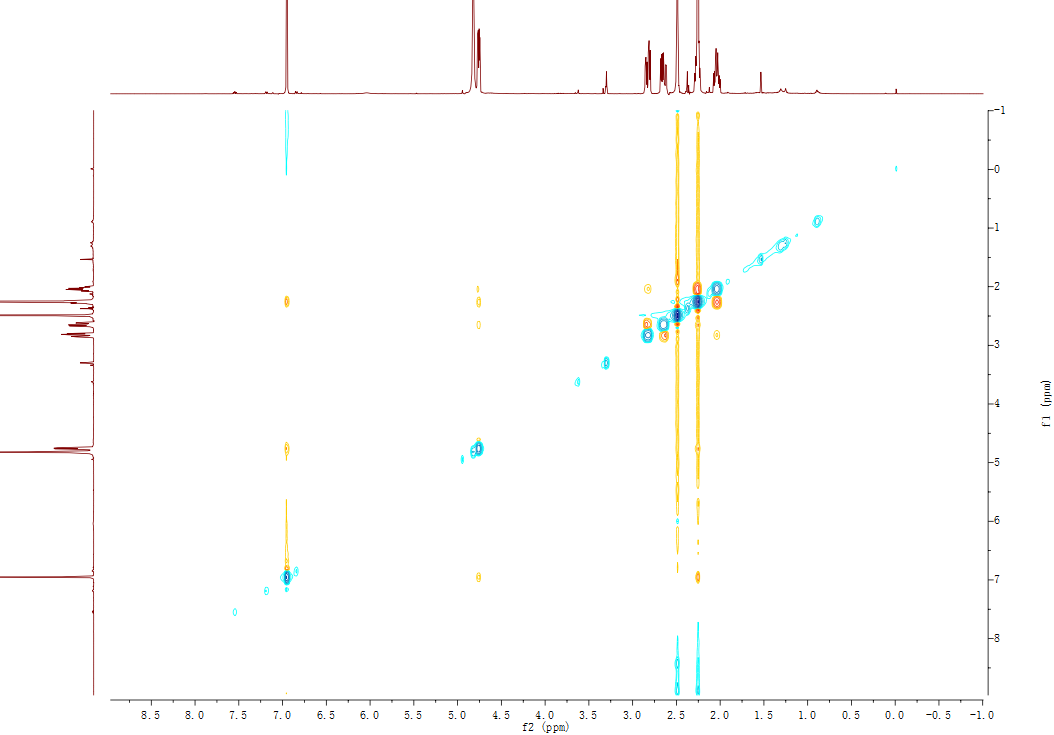


## [**Fig. S34** ROESY spectrum of compound **4** in CD_3_OD](#_Toc61201454)


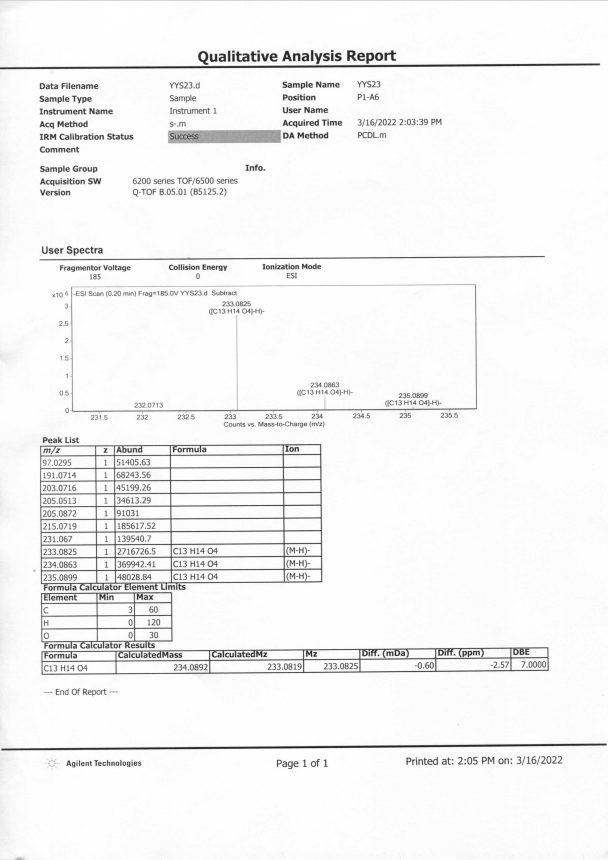


## [[**Fig. S35** The (-)-HRESIMS spectroscopic data of compound **4**](#_Toc61201450)](#_Toc61201456)


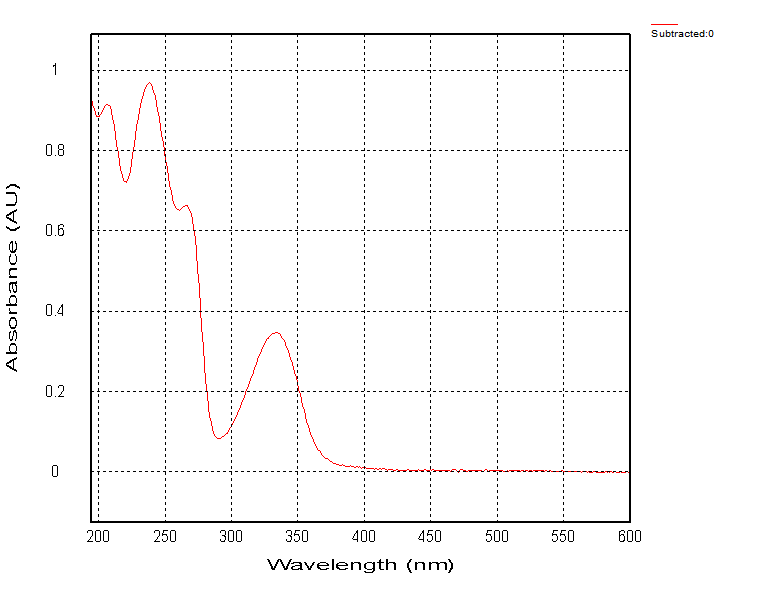


## **Fig. S36** The UV spectrum of compound **4** in CD_3_OD


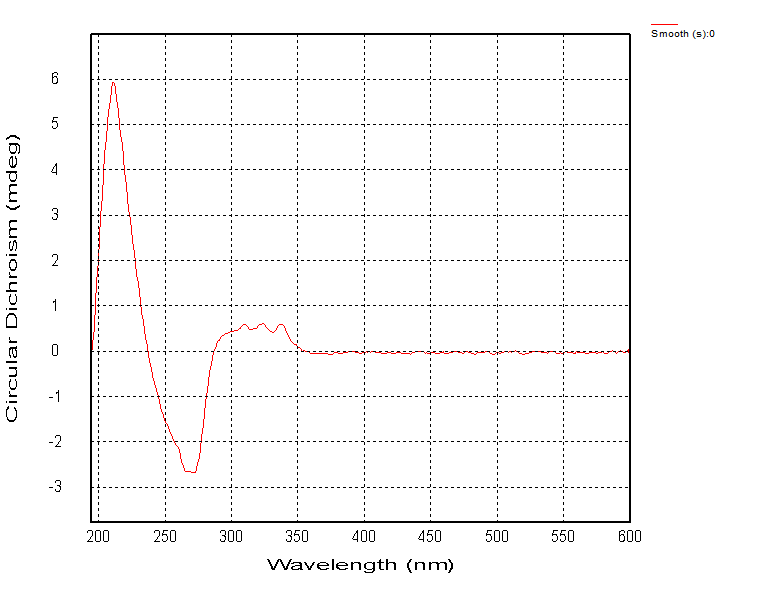


## **Fig. S37** Experimental ECD spectrum of compound **4** in CD3OD


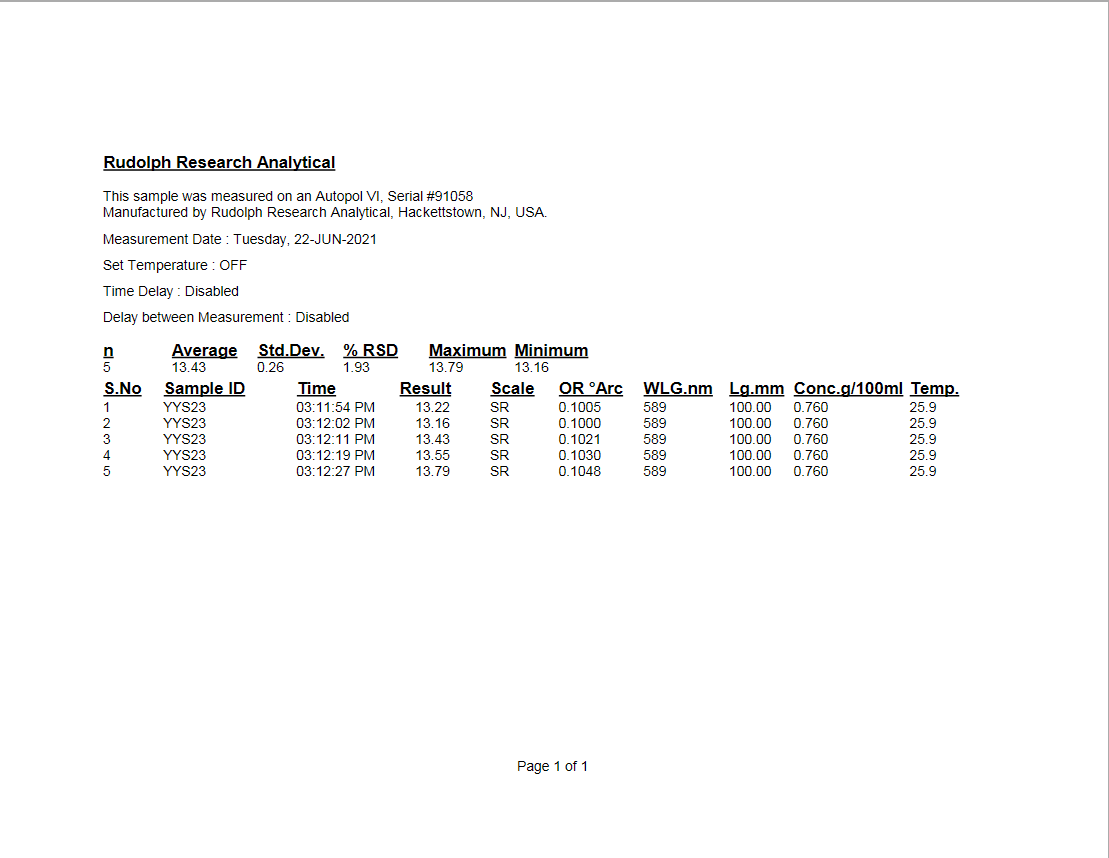


## **Fig. S38** OR of compound **4** in MeOH


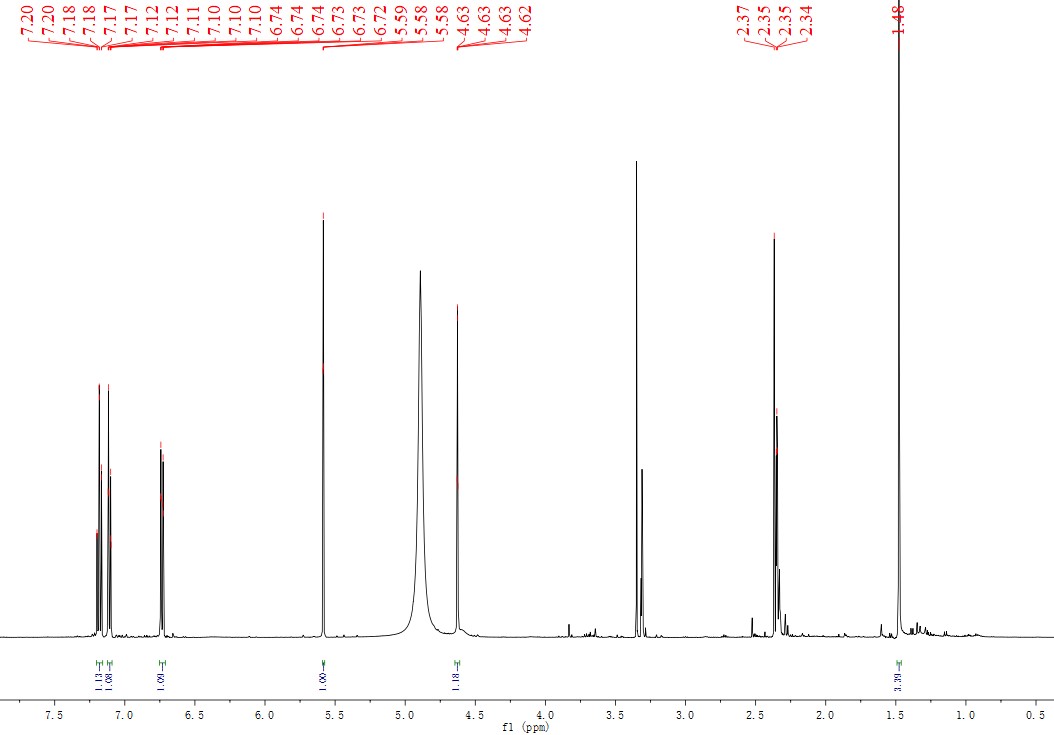


## **Fig. S39** The ^1^H NMR spectrum of compound **5** in CD_3_OD

##
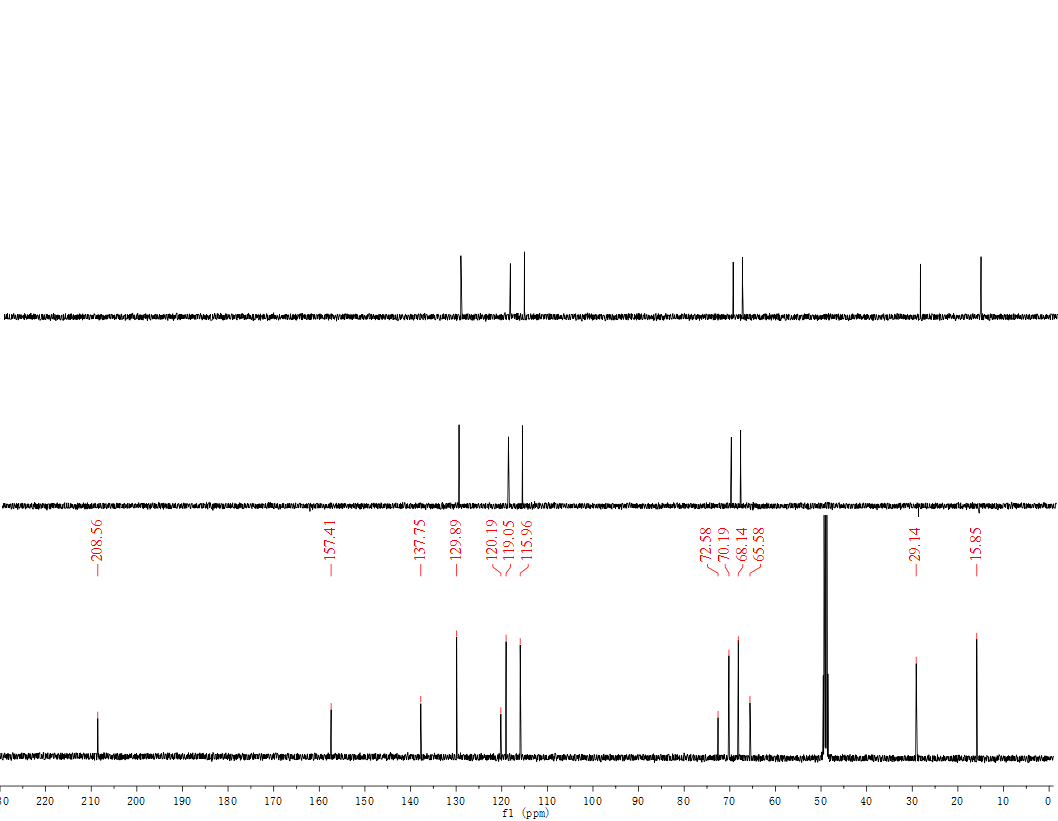


## **Fig. S40** The ^13^C and DEPT NMR spectra of compound **5** in CD_3_OD


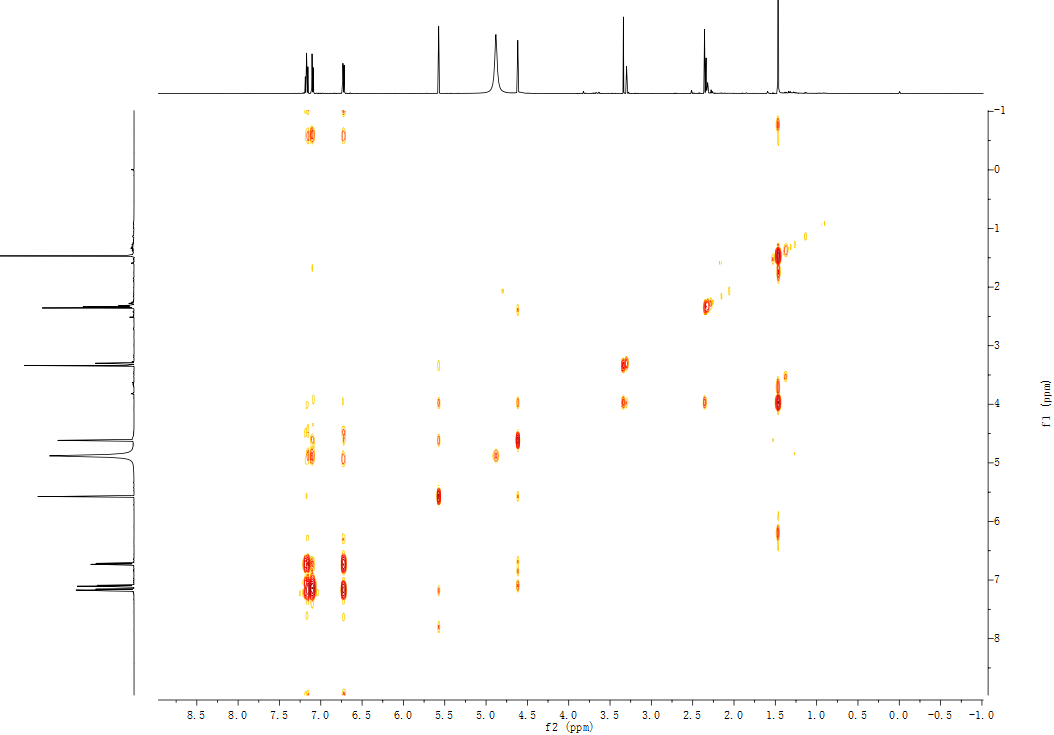


## **Fig. S41** ^1^H-^1^H COSY spectrum of compound **5** in CD_3_OD


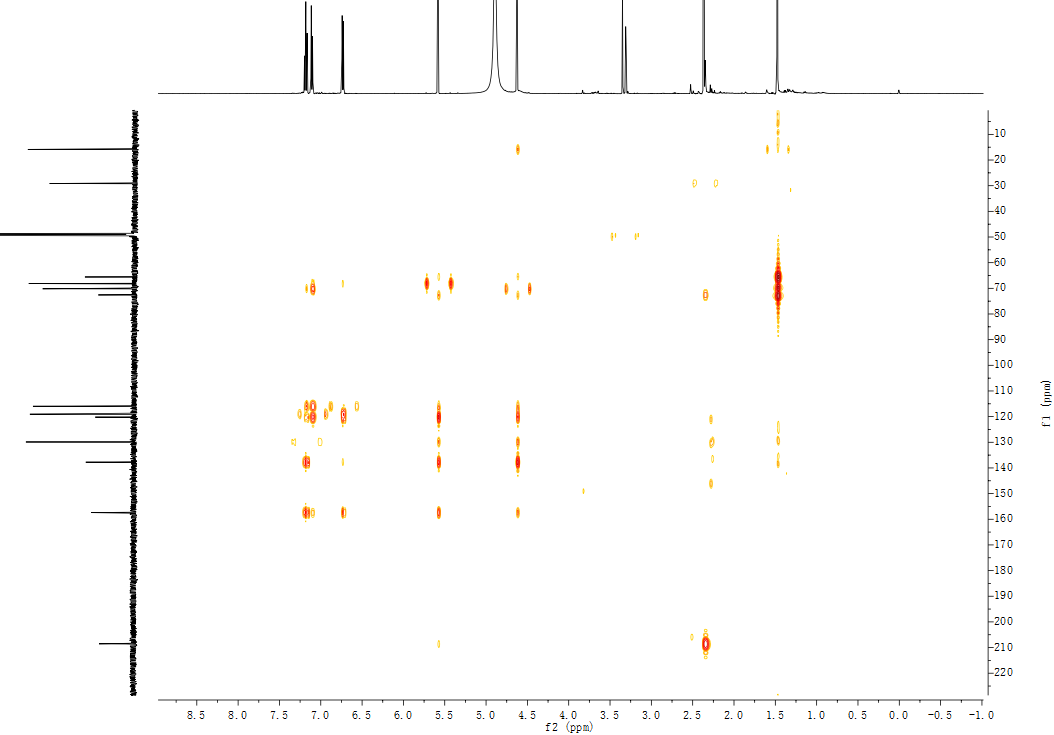


## **Fig. S42** HMBC spectrum of compound **5** in CD_3_OD


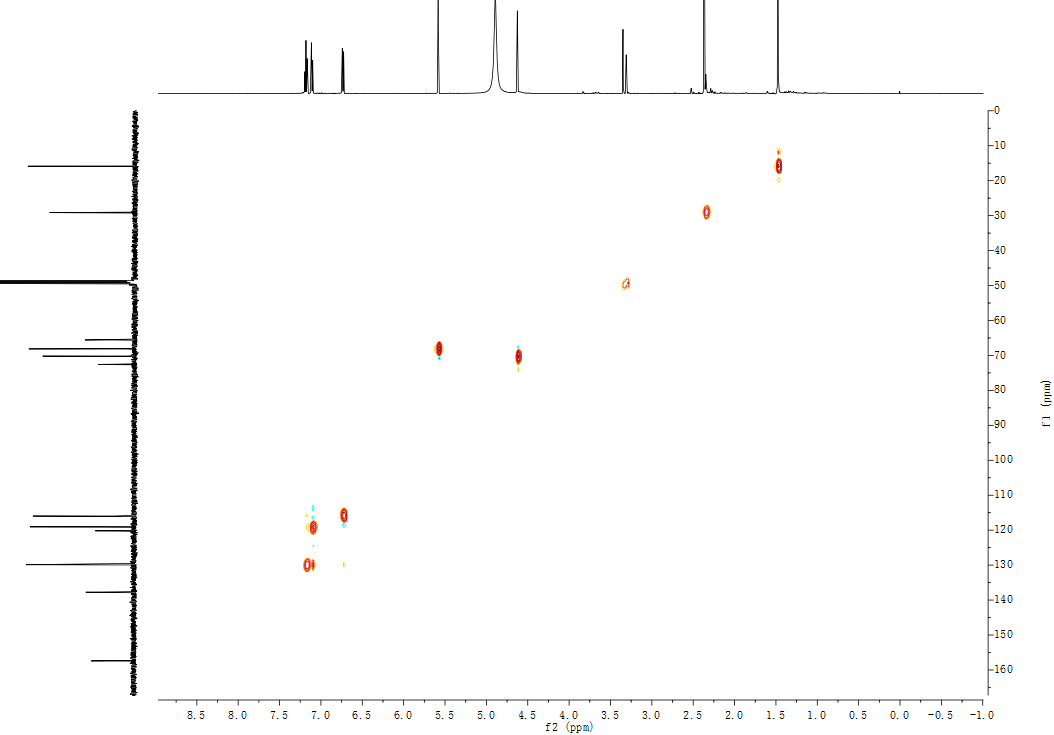


## **Fig. S43** HSQC spectrum of compound **5** in CD_3_OD


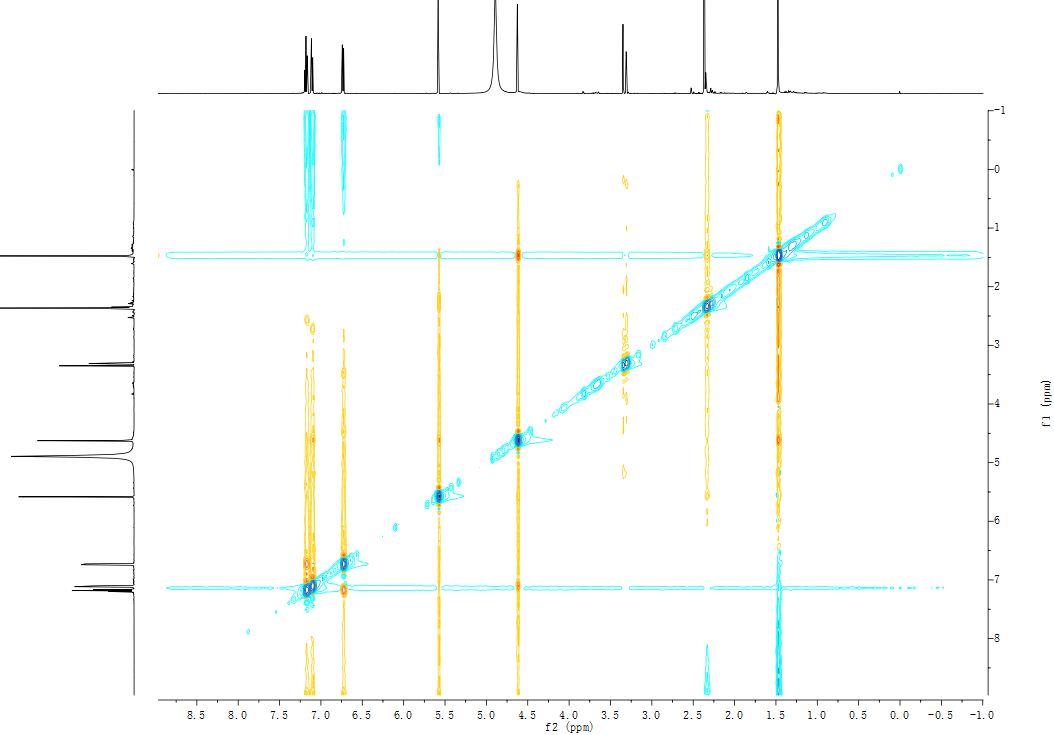


## [**Fig. S44** ROESY spectrum of compound **5** in CD_3_OD](#_Toc61201454)


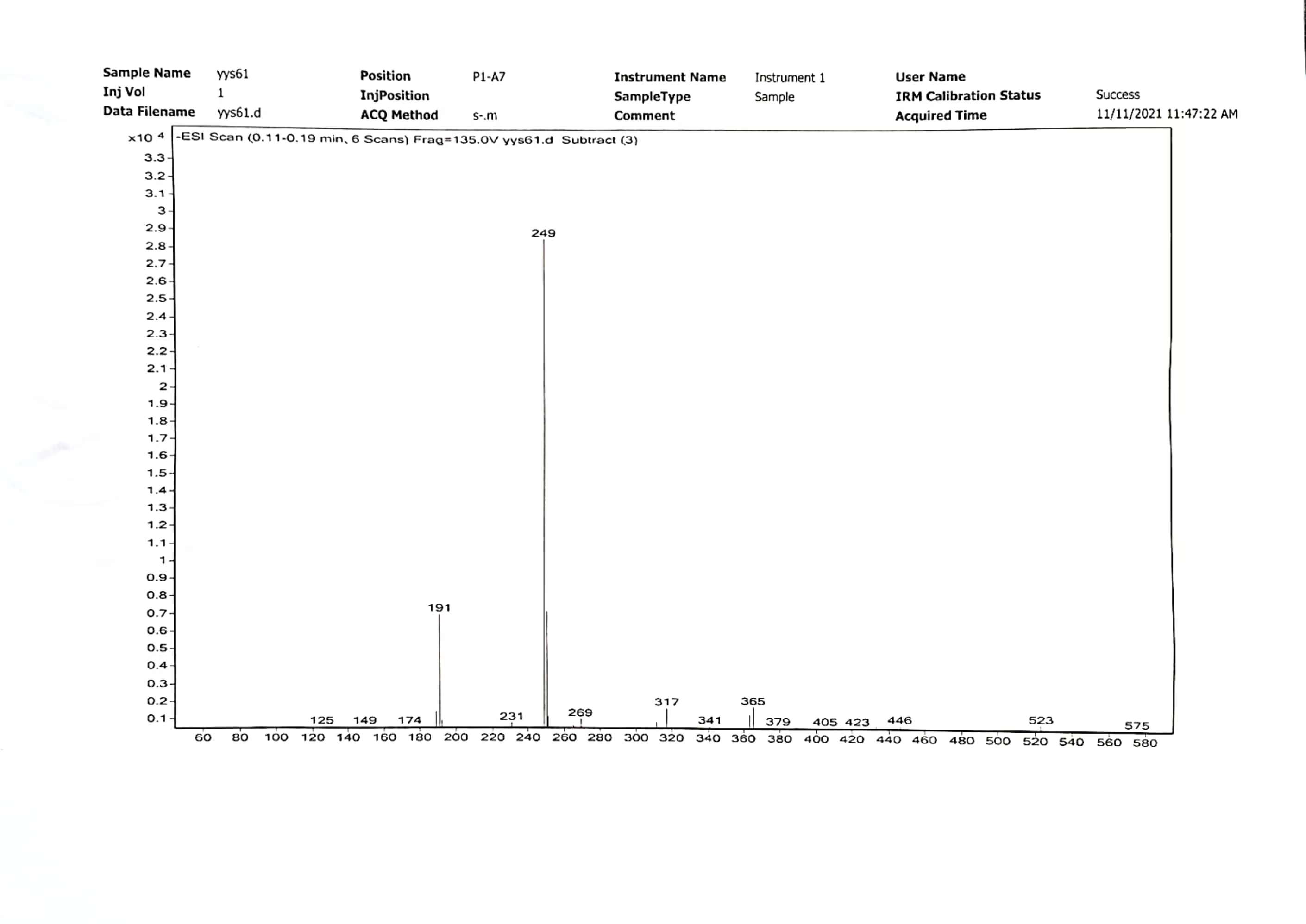


## [[**Fig. S45** The (-)-ESIMS spectroscopic data of compound **5**](#_Toc61201450)](#_Toc61201456)

| Compounds | *Epidermophyton floccosum* | | *Trichophyton rubrum* | *Microsporum gypseum* | |
| --- | --- | --- | --- | --- | --- |
|  | Inhibition rate (%) | MIC_50_ (*μ*M)^c^ | Inhibition rate (%) ^b^ | Inhibition rate (%)^b^ | MIC_50_ (*μ*M)^c^ |
| **1** | 20.478 ± 4.44 | － | 22.002 ± 1.348 | 5.003 ± 2.407 | － |
| **6** | 22.362 ± 2.215 | － | 4.607 ± 0.337 | 3.816 ± 2.183 | － |
| **7** | 15.037 ± 1.604 | － | 16.998 ± 1.685 | 3.183 ± 1.176 | － |
| **8** | 2.272 ± 2.103 | － | -92.137 ± 1.685 | 7.364 ± 1.706 | － |
| **9** | 87.994 ± 0.894 | 2.467 ± 0.03 | 63.777 ± 1.08 | 92.016 ± 1.386 | 4.673 ± 0.077 |
| **10** | 20.696 ± 4.406 | － | 1.935 ± 1.44 | 44.019 ± 3.275 | － |
| **11** | 67.457 ± 3.128 | － | -1.884 ± 4.679 | 20.778 ± 0.63 | － |
| **12** | 41.865 ± 1.788 | － | -1.12 ± 1.44 | 21.669 ± 4.408 | － |
| **13** | 31.754 ± 2.96 | － | 6.261 ± 1.08 | 13.209 ± 1.26 | － |
| **14** | 39.337 ± 1.703 | － | 5.752 ± 1.08 | 28.347 ± 0.756 | － |
| Terbinafine^d^ | 101.264 ± 1.095 | 0.012 ± 1.287 | 101.697 ± 0 | 99.526 ± 0.412 | 0.00258 ± 0.077 |

## **Table S1** Inhibitory effects of **1**, **6**-**14** against three strains skin fungi^a,b^

^a^Data expressed as means ± SD (n = 3); ^b^At a concentration of 100 *μ*M; ^c^ Inhibition rates than 70.0 % were screened for the MIC_50_ values; ^d^Positive control.

## **Table S2** Anti-inflammatory effects of **1**, **6**-**14**

| Compounds | Concentration (*μ*M) | Inhibition rate (%)^a^ |
| --- | --- | --- |
| **1** | 50 | 4.63 ± 0.96 |
| **6** | 50 | 3.52 ± 1.26 |
| **7** | 50 | 4.60 ± 2.43 |
| **8** | 50 | 5.47 ± 1.71 |
| **9** | 50 | 42.88 ± 1.44 |
| **9** | 25 | 21.41 ± 1.77 |
| **10** | 50 | 4.80 ± 2.44 |
| **11** | 50 | 4.20 ± 1.16 |
| **12** | 50 | 20.32 ± 0.25 |
| **13** | 50 | 14.44 ± 1.61 |
| **14** | 50 | 10.85 ± 2.45 |
| L-NMMA^b^ | 50 | 56.08 ± 1.11 |

^a^Data expressed as means ± SD (n = 3). ^b^Positive control.
